# Snf2h Drives Chromatin Remodeling to Prime Upper Layer Cortical Neuron Development

**DOI:** 10.3389/fnmol.2019.00243

**Published:** 2019-10-17

**Authors:** Matías Alvarez-Saavedra, Keqin Yan, Yves De Repentigny, Lukas E. Hashem, Nidhi Chaudary, Shihab Sarwar, Doo Yang, Ilya Ioshikhes, Rashmi Kothary, Teruyoshi Hirayama, Takeshi Yagi, David J. Picketts

**Affiliations:** ^1^Regenerative Medicine Program, Ottawa Hospital Research Institute, Ottawa, ON, Canada; ^2^Department of Cellular and Molecular Medicine, University of Ottawa, Ottawa, ON, Canada; ^3^Departamento de Biología Celular y Molecular, Facultad de Ciencias Biológicas, Pontificia Universidad Católica de Chile, Santiago, Chile; ^4^Departments of Biochemistry, Microbiology and Immunology, University of Ottawa, Ottawa, ON, Canada; ^5^Ottawa Institute of Systems Biology, University of Ottawa, Ottawa, ON, Canada; ^6^KOKORO-Biology Group, Integrated Biology Laboratories, Graduate School of Frontier Biosciences, Osaka University, Suita, Japan; ^7^Department of Anatomy and Developmental Neurobiology, Tokushima University Graduate School of Medical Sciences, Tokushima, Japan

**Keywords:** telencephalon development, chromatin remodeling, ISWI, Smarca5, Snf2h

## Abstract

Alterations in the homeostasis of either cortical progenitor pool, namely the apically located radial glial (RG) cells or the basal intermediate progenitors (IPCs) can severely impair cortical neuron production. Such changes are reflected by microcephaly and are often associated with cognitive defects. Genes encoding epigenetic regulators are a frequent cause of intellectual disability and many have been shown to regulate progenitor cell growth, including our inactivation of the *Smarca1* gene encoding Snf2l, which is one of two *ISWI* mammalian orthologs. Loss of the Snf2l protein resulted in dysregulation of Foxg1 and IPC proliferation leading to macrocephaly. Here we show that inactivation of the closely related *Smarca5* gene encoding the Snf2h chromatin remodeler is necessary for embryonic IPC expansion and subsequent specification of callosal projection neurons. Telencephalon-specific *Smarca5* cKO embryos have impaired cell cycle kinetics and increased cell death, resulting in fewer Tbr2+ and FoxG1+ IPCs by mid-neurogenesis. These deficits give rise to adult mice with a dramatic reduction in Satb2+ upper layer neurons, and partial agenesis of the corpus callosum. Mice survive into adulthood but molecularly display reduced expression of the clustered protocadherin genes that may further contribute to altered dendritic arborization and a hyperactive behavioral phenotype. Our studies provide novel insight into the developmental function of Snf2h-dependent chromatin remodeling processes during brain development.

## Introduction

The proper wiring of the mammalian brain is necessary for cognitive control, including linguistics, motor functions, emotions, memory and associative processing. The six neuronal layers of the cerebral neocortex project directly or indirectly to all brain structures, connect the cerebral hemispheres and provide the neural diversity that is necessary for higher order cognitive skills (Kwan et al., 2012). Indeed, genetic mutations affecting the development and/or function of the neocortex result in a wide array of neurobehavioral alterations and are the cause of numerous intellectual-disability syndromes. This is indicated by the growing number of genes encoding chromatin remodeling proteins as the cause of a wide range of developmental disorders associated with intellectual disability (Rangasamy et al., 2013; Hota and Bruneau, 2016; Sokpor et al., 2017). Nonetheless, how epigenetic programs and chromatin modulators coordinate the proper development and functional maturation of the mammalian brain remains largely uncharacterized.

During murine development, the telencephalon arises from the expansion of a single layer of pseudostratified neuroepithelial cells located in the dorsolateral wall of the ventral neural tube. These cells undergo largely symmetric divisions to expand the progenitor pool but also generate the earliest born neurons (Gotz and Huttner, 2005). Between embryonic day 10 (E10) and E12, the neuroepithelial progenitors transform to radial glia progenitors (RGCs) which reside adjacent to the lateral ventricle in a proliferative layer known as the ventricular zone (VZ) (Kriegstein and Gotz, 2003; Gotz and Huttner, 2005). RGCs are distinguished by their expression of Pax6, Sox9, and the glial proteins GLAST and BLBP (Kriegstein and Gotz, 2003). They extend processes to both the apical/ventricular and basal/pial surfaces and undergo interkinetic nuclear migration to divide at the apical surface (Kriegstein and Gotz, 2003; Gotz and Huttner, 2005). The RGCs can divide symmetrically or asymmetrically to self-renew, generate projection neurons directly, or produce a committed progenitor known as an intermediate progenitor cell (IPC) (Taverna et al., 2014; Hevner, 2019). All IPCs express Tbr2 and Afap1 but can be further subdivided into ventricular IPCs (vIPC) and outer IPCs (oIPC) based on morphology (Hevner, 2019). The vIPCs have a radial bipolar morphology and reside in the VZ where they initially retain contact with the ventricular surface before detaching and becoming oIPCs (Hevner, 2019). The oIPCs are multipolar cells that form the subventricular zone (SVZ) and are enriched in neuronal differentiation markers (Neurod1 and Mgat5b). The IPCs typically divide 1–2 times before differentiating into projection neurons while a minority can produce upwards of 12 neurons (Sessa et al., 2008; Mihalas and Hevner, 2018). While it was originally thought that IPCs only generated upper layer neurons, it is now widely accepted that IPCs produce neurons for all cortical layers (Kowalczyk et al., 2009; Mihalas et al., 2016). Neuronal birthdating experiments demonstrated that the progenitors produce neurons in a temporal sequence with the deeper layer (layer VI and V) neurons generated prior to the upper (layer II/III) neurons (McConnell, 1991). Transplantation experiments determined that the neural progenitors undergo temporal changes to fate competency that result from a combination of extrinsic signals and an intrinsic program (McConnell, 1988). Later experiments have shown that this spatiotemporal sequence is regulated by several transcription factors (e.g., Foxg1, Fezf2) (Molyneaux et al., 2005; Toma et al., 2014), epigenetic mechanisms (Morimoto-Suzki et al., 2014), and a cortical derepression circuit for the proper transition from deep layer (DL) to upper layer (UL) neurogenesis (Srinivasan et al., 2012). Moreover, the precise mechanisms and involvement of epigenetic regulators remain poorly understood and constitute an intense area of investigation (Greig et al., 2013; Toma and Hanashima, 2015).

Gene expression is ultimately regulated at the level of chromatin, where ATP-dependent chromatin remodeling complexes (CRCs) control DNA replication and transcription, DNA accessibility, chromosome structure and ultimately gene expression. More than 30 different genes encode for the catalytic subunits of CRCs in mammals, and multiple DNA-protein binding domains further specify and diversify their strategies to interact with nucleosomes (Narlikar et al., 2013). The *ISWI (Imitation of Swi2/Snf2)* nucleosome remodelers are part of several complexes that catalyze DNA-dependent chromatin remodeling in all eukaryotic species. From *Drosophila melanogaster*, the first ISWI-containing complexes were isolated: NURF (nucleosome-remodeling factor), ACF (chromatin-assembly factor) and CHRAC (chromatin accessibility complex) (reviewed in Erdel and Rippe, 2011; Toto et al., 2014; Goodwin and Picketts, 2018). Mammals possess two *ISWI* orthologs, *Smarca5* and *Smarca1* (encoding Snf2h and Snf2l, respectively), that reside in the conserved *Drosophila* complexes mentioned above, but have also been identified within four additional mammalian-specific complexes, namely three Snf2h containing complexes (NORC, RSF, WICH), and one Snf2l complex (CERF) (LeRoy et al., 1998; Strohner et al., 2001; Bozhenok et al., 2002; Banting et al., 2005). Recently, an *in vitro* study has shown that Snf2h and Snf2l may interchange within these complexes further increasing complexity (Oppikofer et al., 2017). The ISWI protein complexes play significant roles in DNA replication and repair (Aydin et al., 2014), transcriptional regulation (Barak et al., 2003; Lazzaro et al., 2006; Song et al., 2009; Sala et al., 2011; Wiechens et al., 2016), and higher order chromatin structure (Erdel and Rippe, 2011).

The Snf2h and Snf2l proteins have divergent patterns of expression in the mouse embryo suggesting that they have differential roles during development (Lazzaro and Picketts, 2001). Indeed, *Smarca5*-null embryos die during the peri-implantation stage due to hypoproliferation of the inner cell mass and trophoectoderm (Stopka and Skoultchi, 2003), while *Smarca1*-null mice survive normally, but display hyperproliferation of cortical progenitors, resulting in an enlarged brain (Yip et al., 2012). *Smarca5* deletion in the developing cerebellum results in cerebellar hypoplasia and ataxia-like symptoms, while deletion in postmitotic Purkinje neurons results in neural arborization deficits and cognitive alterations (Alvarez-Saavedra et al., 2014). Most recently, *Smarca5* was shown to also mediate lens development and hematopoietic stem cell renewal (He et al., 2016; Kokavec et al., 2017).

While mice inactivated for several partner proteins of Snf2h have been generated, a clear role in neocortical development for Snf2h or Snf2h-containing CRCs remains largely unexamined (Banting et al., 2005; Zaghlool et al., 2016; Goodwin and Picketts, 2018). We have shown that *Smarca1* encoding Snf2l controls cell cycle exit through FoxG1 dosage to modulate neural output and cortical differentiation (Yip et al., 2012). We therefore interrogated the role of Snf2h during neocortical development by conditional deletion of the *Smarca5* gene in the mouse. We show that Snf2h ablation alters cell cycle kinetics and reduces Tbr2^+^ and FoxG1^+^ neuroprogenitor expansion. These deficits largely result in reduced production of upper layer neurons. Furthermore, Snf2h mediates callosal neuron projections as we observed altered expression of the clustered *protocadherin-*β genes and altered targeting of axons, that contribute to partial agenesis of the corpus callosum. Taken together, our studies indicate multiple roles for Snf2h in the developing neocortex and suggest that it could be a key contributor to autism related disorders.

## Materials and Methods

### Mouse Breeding

The generation of the *Snf2h*^*fl/fl*^ mice have been described previously (Stopka and Skoultchi, 2003; Alvarez-Saavedra et al., 2014). *Snf2h*^*fl/fl*^ mice were backcrossed for 6 generations to a C57Bl/6 background and bred with C57Bl/6 Emx1-Cre^–/+^ transgenic line (Gorski et al., 2002) that also carried a *Snf2h* null allele (Stopka and Skoultchi, 2003), thereby generating *Snf2h* cKO mice by Emx1-Cre (*Snf2h*^–/fl^:Emx1-Cre^–/+^) and control littermates that carried only one functional copy of *Snf2h* (*Snf2h*^–/fl^:Cre^–/–^ or *Snf2h*^+/fl^:Cre^–/+^) or both functional alleles (*Snf2h*^+/fl^:Cre^–/–^). These three control genotypes showed no overt phenotypes, reproduced normally and lived into adulthood and were used interchangeably as controls, except where indicated. For embryo staging, embryonic day 0.5 (E0.5) was defined as noon of the day a vaginal plug was observed after overnight mating. Animals were kept in an animal house under SPF (specific pathogen-free) conditions in a 12 h/12 h light:dark cycle with water and food *ad libitum.* All animal experiments were approved by the University of Ottawa’s Animal Care ethics committee, with the guidelines set out by the Canadian Council on Animal Care.

C57Bl/6 wild type mice were purchased from Charles River (Montreal, QC, Canada).

### Behavioral Analysis

All behavioral tests were completed in the Behavior Core Facility at the University of Ottawa using standardized protocols. Animals were habituated to the testing room at least 1 h before testing. Female and male mice were assessed independently at 4–6 months old, for which we did not observe sex-specific differences in behavior and hence the data was pooled. For behavioral assays, one-way ANOVA was used for at least 7–10 mice per genotype. The values are presented as the mean ± SEM.

#### Morris Water Maze

The water maze pool was maintained at 22 ± 1°C. A white platform was submerged 1 cm below the water’s surface in the center of the target quadrant. Mice were randomly placed on one of the starting points in one of four quadrants and given 60 s to find the hidden platform. Mice that did not find the platform at the end of the 60 s period were manually guided to the platform and allowed to rest for 20 s. Each mouse had four trials per day for 10 consecutive days. An animal was scored as successfully finding the platform if it succeeded in two of four trials per day. In the visual assays, mice were placed on one of four quadrants and allowed to find the visible platform in 60 s. The swimming path of mice was recorded by an automated video camera and analyzed by the Ethovision 7 XT (Wageningen, Netherlands).

#### Open Field

Animals were placed in the center of a 45 × 45 × 45-cm chamber equipped with photobeams (Accuscan) to record activity during a 10-min test period.

#### Elevated Plus Maze

Animals were habituated to the test room for at least 2 days prior to test. Animals were placed in the center of a maze consisting of two arms (each arm 5 cm wide × 60 cm long) enclosed by ∼15 cm high walls, and two open arms (each arm 25 × 7.5 cm, with a raised 0.5 cm lip at edges) elevated 1 m above ground and with equidistant arms from the center of the platform. The amount of time the animals spent in the open or closed arms, the total number of entries and the total distance traveled were recorded for 10 min using video detection software (Ethovision 7 XT, Wageningen, Netherlands).

#### Social Interactions

A control mouse is placed in the corner of an open field box (under a red light) that measures 45 cm long on each side × 45 cm high and containing a 5.5 × 9.6 cm wire mesh rectangular cage. The mouse is given 5 min to explore the arena and then removed. A few seconds later, a test mouse (or social target), of the same strain, age and gender is placed inside the rectangular wire mesh cage and the control mouse placed back in the arena. The time the social target interacts with the control mouse in 5 min trials is recorded using Ethovision 7 XT automatic tracking software. Total distance traveled, time spent in 2 corners across the wire mesh cage and velocity is also recorded.

#### Fear Conditioning

On the first day (training), the animal is placed in the fear conditioning apparatus for a total of 6 min. After the first 2 min in the apparatus a tone is played for 30 s ending with a 2-s shock. One minute following the shock, the tone is played again for 30 s ending with a 2-s foot shock. For the remaining 2 min there is no tone or shock. The freezing behavior of the animal is recorded throughout the 6 min. This is the training in which the mouse receives 2 exposures to the tone followed by the shock and this occurs in a novel context, which is the conditioning box. On the 2nd day, contextual conditioned fear testing begins. This measures the fear associated with being in the same environment where the shock was delivered (done ∼24 h after training). The mouse is placed in the same apparatus with all the same lighting and room conditionings for 6 min and freezing behavior is recorded.

### EdU- and BrdU-Labeling

Timed-pregnant females were injected intraperitoneally with 100 μg/g body weight of 5-bromo-2′-deoxyuridine (BrdU; Sigma-Aldrich, Oakville, ON, Canada) or of 5-Ethynyl-2′-deoxyuridine (EdU; Sigma-Aldrich) and embryos or pups killed at the indicated times. For EdU-pulse labeling, animals were sacrificed 60 min later, embryos quickly dissected, and fixed in 4% PFA overnight. The following day, the brain tissue was submerged in a 1:1 solution of 30% sucrose solution and OCT (Tissue-Tek, Sakura Americas, Torrance, CA, United States), snap-frozen on liquid nitrogen and stored at −80°C. For embryos, 12 μm sections were obtained using a Leica CM1850 cryostat. For EdU immunodetection, sections were first washed with 1X PBS prior to incubation with an EdU staining solution (100 mM Tris-HCL pH 7.2, 2 mM CuSO_4_, 10 μM fluorescent azide, 50 mM ascorbic acid) for 1 h at room temperature. Slides were then washed several times with 1X PBS, incubated with Hoechst 33342 dye (2′-[4-ethoxyphenyl]-5-[4-methyl-1-piperazinyl]-2,5′-bi-1H-benzimidazole trihydrochloride trihydrate; Thermo Fisher Scientific, Waltham, MA, United States) for 15 min at RT and mounted with DAKO fluorescent mounting medium (Agilent Technologies, Santa Clara, CA, United States). For BrdU-birthdating of upper-layer cortical neurons, pregnant dams were injected with a single BrdU dose (100 μg/g body weight) at E13.5 and pups killed at birth. For BrdU immunodetection, sections were incubated in 2N HCl for 10 min at 37°C, rinsed in 0.1 M sodium borate, pH 8.3, blocked and incubated overnight at 4°C with rat monoclonal anti-BrdU antibody (1:300; Abcam #6326). A cell was considered BrdU+ if >75% of the nuclei was stained. The average number of immunopositive cells was determined from five separate fields under ×40 magnification in confocal Z-stacks (or cubic bins) of 18 × 10^3^ μm^3^.

For cell cycle length experiments, BrdU (100 μg/g body weight) was injected into pregnant dams at E13.5, E14.5, and E15.5 followed by EdU injection 1.5 h later. Pups were sacrificed 30 min after EdU administration, brains dissected and fixed in 4% PFA overnight. Sections were immunostained with BrdU and EdU as described above. Two sections per animal from three different animals per genotype were scored and cell cycle length was estimated using the following calculations as described previously (Martynoga et al., 2005; Quinn et al., 2007). Double labeled cells (EdU+, BrdU+) represent the fraction in S-phase (S_*cells*_), whereas the leaving fraction is the proportion of cells that were BrdU+, EdU− (L_*cells*_) and DAPI+ cells within the VZ/SVZ represent the entire pool (P_*cells*_). Time in S-phase (T_*S*_) is calculated as T_*S*_ = S_*cells*_/L_*cells*_ x 1.5 h. Cell cycle length (T_*C*_) was calculated T_*C*_ = P_*cells*_/S_*cells*_xT_*S*_. Finally, T_*C*_ values were normalized to the control sample and statistics performed as described below.

### TUNEL Assay

Sections were examined for DNA fragmentation with the TUNEL *in situ* cell death detection kit (Roche Applied Science, ON, Canada) according to the manufacturer’s instructions. The average number of TUNEL-positive cells was determined from five separate fields under ×40 magnification in cubic bins as described above.

### Histology

**FIGURE 1:**
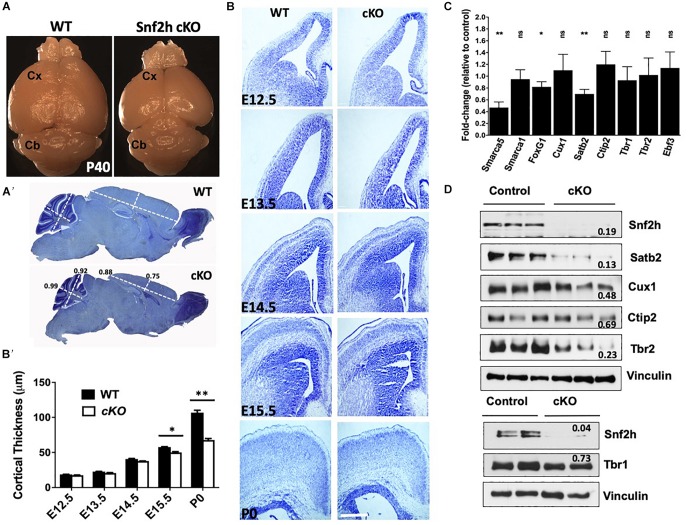
A reduced neocortex is observed in Snf2h cKO mice. **(A)** Adult brains (P40) from *Snf2h* cKO mice show a gross reduction in the size of the neocortex (Cx) compared to WT littermates, but no apparent difference in the size of the cerebellum (Cb). **(A’)** Cresyl violet stained midline sagittal sections of P60 WT and *Snf2h* cKO brains highlight the reduced size of the mutant cortex. Relative thickness differences are shown above the dotted lines that depict the location of dorsal-ventral (D-V) and anterior-posterior (A-P) positions used for analysis. **(B)** Cresyl violet stained coronal sections from WT and *Snf2h* cKO brains throughout neurogenesis (E12.5-E15.5) and at birth (P0). Scale bar, 100 μm. **(B’)** Quantification of cortical thickness at different ages indicates that the reduced size of the *Snf2h* cKO neocortex becomes apparent at E15.5. **(C)** Normalized RT-qPCR expression of the ISWI genes (*Smarca5* and *Smarca1l*), and key transcription factors involved in neocortical development (*FoxG1, Cux1, Satb2, Ctip2, Tbr1, Tbr2*, and *Ebf3*) from mutant cortical samples relative to WT samples at E15.5. Mean values were used to calculate the fold change differences relative to control levels. ^∗^*P* < 0.05, ^∗∗^*P* < 0.01, *n* = 4, Student’s *t*-test. **(D)** Immunoblots for cortical layer markers in neocortical extracts prepared from E15.5 brains. Vinculin was used as loading control. Values denote averaged pixel densitometry normalized to Vinculin and relative to control samples, *n* = 3 mice per genotype.

Nuclei of brain sections were visualized using cresyl violet staining in 20–40 μm frozen sections. Sections were cleared in Xylene and mounted with Permount. Brain measurements were performed using ImageJ software^1^ with a minimum of three animals per genotype. Average cortical thickness was determined for different embryonic ages or adult brains and plotted directly (Figure 1B’) or relative to wild type (Figure 1A’ and Supplementary Figure 2).

### Golgi-Cox Staining

Golgi staining was performed using FD Rapid GolgiStain Kit (FD NeuroTechnologies, Columbia, MD, United States). Briefly, P180-P240 mice were intracardially perfused with 4% paraformaldehyde in 0.1M PBS and brains quickly dissected. Tissues were sectioned at 100 μm, mounted on gelatin-coated slides, and further stained according to the manufacturer’s instructions. Cortical neuron morphology was analyzed using ImageJ software^2^. A minimum of 10 neurons per section per genotype were analyzed.

### Immunofluorescent Histochemistry

For postnatal brains, 40–50 μm free-floating sections were used. Sections were washed four times in PBST (PBS with 0.1% Triton X-100), blocked (1 h, room temperature) in 10% horse serum/PBST, and incubated (overnight, 4°C) in primary antibodies. The following primary antibodies were used at 1:200, unless indicated: rabbit anti-Ki67 (Abcam #72499); rabbit anti-Tbr2 (Abcam #37003); rat anti-BrdU (Abcam #6326); rabbit anti-FoxG1 (Abcam #18259); mouse anti-SATB2 (1:50; Abcam #51502); rabbit anti-Pax6 (1:100, Abcam #195045); rabbit anti-Brn2 (Abcam #94977); rabbit anti-FoxP1 (Abcam #16645); rat anti-Ctip2 (#18465); rabbit anti-Tbr1 (Abcam #31940); rabbit anti-Snf2h (Bethyl Laboratories #A301-017A); mouse anti-BrdU (1:100, DAKO); rabbit anti-phospho histone H3 (Millipore #06-570); rabbit anti-Cux1 (1:50; Santa Cruz sc-6327); rabbit anti-γH2AX (Cell Signaling Technology #9718); rabbit anti-CC3 (cleaved caspase-3; Cell Signaling Technology #9661); mouse anti-Calbindin (Sigma-Aldrich #C9848); mouse anti-NeuN (1:500, EMD Millipore #MAB377); and mouse anti-MAG (1:500; EMD Millipore #MAB1567). The following day, sections were washed five times in PBST and incubated (2 h, room temperature) with DyLight^488^, DyLight^594^ or DyLight^649^-conjugated mouse pre-adsorbed secondary antibodies (1:1000, Jackson ImmunoResearch, West Grove, PA, United States) against the IgG domains of the primary antibodies. All sections were counterstained with Hoescht 33342 dye (Thermo Fisher Scientific) and mounted on slides with DAKO Fluorescent mounting medium (Agilent Technologies).

### DiI/DiA Axonal Tracing

P7 and P21 brains from Snf2h cKO-Emx1 mice and control littermates (*n* = 4 mice per genotype) were dissected in PBS and fixed in 4% PFA for 3 days at 4°C. Each brain was placed in a Petri dish and observed under a stereomicroscope to determine the specific dye placement location based on the description of Gurung and Fritzsch (2004). A small hole was made for each insertion site using a small needle, then using an insertion pin (#26007-02 and #26016-12, Fine Science Tool, Foster City, CA, United States) a single DiA crystal (D3911, Life Technologies, Carlsbad, CA, United States) was inserted in the right motor cortex area. Following the same procedure with a new insert pin, a single Dil crystal (D3883, Life Technologies) was inserted in the right somatosensory cortex area as shown in Figure 5. Each brain was kept in the darkness in 4% PFA at 37°C for a period of incubation of 4 weeks (for P7 brains) or 6 weeks (for P21 brains). Following this incubation period, each brain was transferred to 30% sucrose in PBS for 48–72 h and frozen in OCT. Samples were snap-frozen using liquid nitrogen and kept at −80°C until sectioning. Sagittal cryosections (60 mm) were prepared and gently transferred with a brush into a 24-well plate containing PBS. Sections were washed two times in 1X PBS and incubated for 15 min with Hoescht 33342 dye (Thermo Fisher Scientific). Sections were further rinsed in 1X PBS and mounted with DAKO Fluorescent mounting medium (Agilent Technologies).

### Cell Counts

Cell counts were performed on 3–5 sections per animal and a minimum of four mice per genotype were used. Counts are expressed as a percentage of total DAPI cells unless stated otherwise. For progenitor and cortical layer cell counts, WT and mutant coronal sections were first matched using age-appropriate, specific brain landmarks located outside of the cortex. Following immunostaining, an identical sized box was oriented over the dorsomedial region of the telencephalon within which DAPI+ and marker+ cells were counted for all genotypes. EdU+ cell counts are plotted as a percentage of DAPI+ nuclei located within the VZ/SVZ cells only. Phospho-histone H3 counts are absolute numbers of PH3+ cells spanning 410 μm along the lateral ventricle.

### *In situ* Hybridization

Brains were rapidly removed and embedded in OCT compound and quickly frozen in isopentane cooled with liquid nitrogen. Ten μm sections were obtained through the sagittal brain and hybridization was performed as described previously (Schaeren-Wiemers and Gerfin-Moser, 1993; Noguchi et al., 2009). Digoxigenin (DIG)-labeled RNA probes were synthesized from cDNA clones using the DIG RNA Labeling Mix (Roche) and with previously published probes (Hirayama and Yagi, 2013).

### Western Blotting

Cortices were quickly dissected from individual embryos and snap frozen in dry ice. Cortices were then homogenized in ice-cold RIPA buffer (10 mM Tris-Cl, pH8.0, 1 mM EDTA, 1% Triton X-100, 0.1% sodium deoxychlorate, 0.1% SDS, 140 mM NaCl, and 1 mM PMSF) supplemented with protease inhibitor cocktail (Sigma-Aldrich) and incubated for 20 min on ice with gentle mixing. After pre-clearing by centrifugation (15 min at 17,000 × *g*), proteins were quantified by the Bradford method. Protein samples were resolved on sodium dodecyl sulfate polyacrylamide gels under denaturing conditions or using Bis-Tris 4–12% and Tris-Acetate 3–8% gradient gels (NuPage, Invitrogen) and blotted onto PVDF membranes (Immobilon-P; Millipore, Burlington, MA, United States) by wet transfer for 1–2 h at 90 V. Membranes were blocked (45 min, room temperature) with 5% skim milk in TBST (Tris-buffered saline containing 0.05% Triton X-100), and incubated (4°C, overnight) with the following antibodies: rabbit anti-Snf2h (1:4000; Abcam #72499); sheep anti-Snf2l (1:2000) (Barak et al., 2003); rabbit anti-FoxG1 (1:1000; Abcam #18259); rabbit anti-Cux1 (1:1000; Santa Cruz sc-6327); rabbit anti-Tbr2 (1:1000; Abcam #37003); mouse anti-Satb2 (1:500; Abcam #51502); rat anti-Ctip2 (1:2000; #18465); rabbit anti-Tbr1 (1:1000; Abcam #31940); rabbit anti-γH2AX (Cell Signaling Technology #9718); rabbit anti-pATM (1:2000; Cell Signaling Technology #13050); mouse anti-ß-Actin (1:30,000; Sigma); and mouse anti-Vinculin (1:10,000; Sigma-Aldrich #V9131). Membranes were incubated (1 h, room temperature) with ImmunoPure^®^ HRP-conjugated goat anti-rabbit or goat anti-mouse IgG (H + L) secondary antibodies (1:30,000; Pierce, Rockford, IL, United States). Membranes were washed 5 × 5 min in TBST after antibody incubations, and signals were detected using the Pierce Supersignal West Fempto chemiluminescence substrate (Cat # 34095). Western blots were quantitated using ImageJ software. At least 2 separate gels were immunoblotted with cortical extracts from independent litters and used for quantitation.

### Reverse Transcription

Embryonic cortices were dissected from mutant and control littermates and RNA was isolated using Trizol (Thermo Fisher Scientific) according to the manufacturer’s instructions. Glycogen (Ambion, Inc., Austin, TX, United States) was used as carrier. One μg of total RNA was reverse-transcribed using SuperScriptIII (Thermo Fisher Scientific), and synthesized cDNA was further diluted 1:25.

### Quantitative Real-Time PCR

qPCR analysis was carried out using the SYBR Green Advantage qPCR premix (Clontech #639676) under the following conditions: one cycle at 95°C for 1 min, and then 40 consecutive cycles at 95°C for 10 s, 60°C for 10 s, and 72°C for 20 s. All primers were analyzed by melt curve analysis after qPCR amplification. The ΔΔCt method was used to compare fold-change. L32 and 18S mRNAs were used as normalizers in separate experiments. Triplicate or quadruplicate samples were performed per reaction and a minimum of 3 mice analyzed per genotype. Student’s *t*-test was used for statistical significance. All qPCR primers are listed in Supplementary Table 1.

### Microarrays

Gene expression profiling was performed on RNA isolated from P0 cortical extracts from *Snf2h* cKO-Emx1 and control littermates. Briefly, newborn pups were decapitated and the cortices quickly dissected from mutant and control littermates. Cortices from a single animal were washed with PBS and then minced with scissors prior to RNA isolation using Trizol (Invitrogen) according to the manufacturer’s instructions. Three cortices were pooled per sample, *n* = 2 per genotype. RNA samples were sent to the Genome Quebec Innovation Centre (Montreal, QC, Canada) for hybridization onto Affymetrix Mouse Gene 1.0 ST microarrays. The microarray data were normalized using robust multi-array average (RMA) with Affymetrix Power Tool and FlexArray 1.6. The output was summarized by gene level and log transformed. Differentially expressed genes were analyzed by Significance Analysis of Microarray (SAM). Genes were scored as differentially expressed on an array if it demonstrated a *P*-value < 0.01, and had sufficient detectable signals across all replicates [A value (log_2_) > 7]. All raw and processed data has been deposited into the GEO database (GSE59152).

### Image Acquisition and Processing

Tissue sections were examined and images captured using a Zeiss 510 laser scanning confocal microscope with UV (405 nm), argon (488 nm), helium/neon (546 nm), and helium/neon (633 nm) lasers. All images were acquired as 10–30 μm Z-stacks (in 1–2 μm intervals) and analyzed as projections using the LSM 510 Image Browser software (Zeiss, Oberkochen, Germany). Epifluorescent and light microscopy images were acquired with a Zeiss Axiovert Observer Z1 epifluorescent/light microscope equipped with an AxioCam cooled-color camera (Zeiss). Images were exported to Adobe Photoshop CS5 (Adobe Systems Inc., San Jose, CA, United States) and further processed for contrast when necessary.

### Magnetic Resonance Imaging

MRI was performed with a General Electric/Agilent MR901 7T small animal MRI scanner using a 72 mm inner diameter radiofrequency transmit coil and mouse brain surface receive coil (Rapid MR International, Columbus, OH, United States). Axial, coronal, and sagittal images were acquired with a 2D T2-weighted fast spin echo pulse sequence with the following parameters: echo time = 25 ms, repetition time = 3 s, echo train length = 8, bandwidth = 15.6 kHz, field of view = 2 cm; slice thickness = 0.7 mm, matrix size = 256 × 256 × 15, number of averages = 6–8, scan time per orientation = 9.5–13 min, in-plane spatial resolution = 78 microns. Three mice were scanned per genotype.

### Statistics

Group statistical analysis was performed via the two-tailed Student’s *t*-test, except for behavioral experiments where one-way ANOVA or two-way ANOVA with multiple comparisons was used. *P* < 0.05 was accepted as statistically significant. A minimum of 3 mice from each genotype were used for evaluation except for behavior studies where 7–10 animals were used per group. The values are presented as the mean ± SEM.

## Results

### Smarca5 Deletion in the Dorsal Telencephalon Alters Progenitor Cell Cycle Kinetics

To investigate the role of Snf2h in the developing neocortex, we utilized the forebrain-specific Emx1-Cre driver mouse line to ablate *Smarca5* gene expression (Gorski et al., 2002). We validated the spatiotemporal specificity of the Emx1-Cre line by first crossing it to the ROSA-STOP-lacZ reporter line. As expected, lacZ was activated at ∼E10.5 specifically in the dorsal telencephalon of reporter mice (Supplementary Figure 1A). In contrast to our previous studies with a Nestin-Cre driver (Alvarez-Saavedra et al., 2014), the inactivation of *Smarca5* using the Emx1-Cre driver resulted in healthy viable animals with a normal lifespan (hereafter referred to as *Snf2h* cKO mice). However, upon dissection the cerebral cortex of *Snf2h* cKO adult mice was visibly smaller than control animals, which is often indicative of a developmental growth defect (Figure 1A and Supplementary Figure 1B).

To quantify this change we imaged cresyl violet or DAPI-stained P60 sagittal sections and measured the thickness of the cortex and cerebellum in dorsal-ventral and anterior-posterior planes. Relative differences indicated that the mutant cortex was reduced by 15–25%, while the cerebellum was only slightly reduced in size (Figure 1A’ and Supplementary Figure 2). The cerebellum showed robust expression of Snf2h which is in stark contrast to the *Snf2h* cKO mouse model generated with the Nestin-Cre driver that showed a dramatic reduction in cerebellar size and lack of Snf2h expression (Supplementary Figures 2, 3A,C) (Alvarez-Saavedra et al., 2014). Since the olfactory bulb is also known to express Emx1 (Gorski et al., 2002), we examined this structure in adult WT and mutant animals confirming that it was reduced in size (WT, 1216 μm ± 47 μm; cKO, 801 μm ± 61 μm), although it retained significant Snf2h expression (Supplementary Figures 3A,B). To determine when the mutant cortex began to show a reduction in size, we analyzed cresyl violet stained coronal sections from brains isolated at E12.5 through to P0 (Figure 1B). Cortical thickness measurements indicated that the size difference became apparent after E14.5 (Figure 1B’).

Cortical projection neurons are generated from radial glial cells (RGCs) and basal/intermediate progenitor cells (IPCs) that reside and expand within the ventricular zone (VZ). Temporal changes within the progenitor pool results in the sequential generation of six cortical neuronal sub-types that populate the different layers, with a general switch from deep layer neuron production to UL neuron production occurring around E14.5 (Adnani et al., 2018). As such, RNA and protein lysates were generated from E15.5 cortices to examine the expression of different markers as an initial screen to indicate specific defects. In this regard we examined the expression of genes encoding markers of the earliest born Cajal-Retzius neurons (*Early B-cell factor*, *Ebf3*), an IPC marker (*Tbr2*), an early marker of neurogenesis (*FoxG1*), and transcription factors characteristic of both DLs (*Tbr1* and *Ctip2*) and ULs (*Cux1* and *Satb2*) (Siegenthaler et al., 2007; Alcamo et al., 2008; Britanova et al., 2008; Chiara et al., 2012; Srinivasan et al., 2012). RT-qPCR experiments revealed that *Smarca5* expression was reduced in the E15.5 mutant cortex by ∼50% (Figure 1C). By comparison, expression of *Smarca1* was unaffected in the mutant cortex relative to controls (Figure 1C). *FoxG1* and *Satb2* mRNA levels were significantly reduced, while the expression levels of all other transcription factors were unaffected (Figure 1C). Similarly, we examined the protein expression of Snf2h, Snf2l and FoxG1 at E15.5. Consistent with the RNA results, we observed reduced protein levels for Snf2h and FoxG1, while Snf2l protein levels were elevated by ∼2-fold (Supplementary Figures 1C,D). Further analysis by immunoblots highlights that the relative expression of both DL (Ctip2, Tbr2) and UL (Cux1, Satb2) neuronal markers was reduced, albeit the UL neurons showed a much larger overall reduction in protein levels than the DL markers (Figure 1D). Moreover, these changes were reflective of *Smarca5* ablation since P0 sections stained with Snf2h antibodies showed a dramatic reduction in protein expression within all cortical layers (Supplementary Figure 1E). Altered protein levels of both DL and UL neuronal markers, particularly with a greater loss of the later-born UL neurons suggests that the reduced cortical size may be caused from a progenitor cell defect.

**FIGURE 2:**
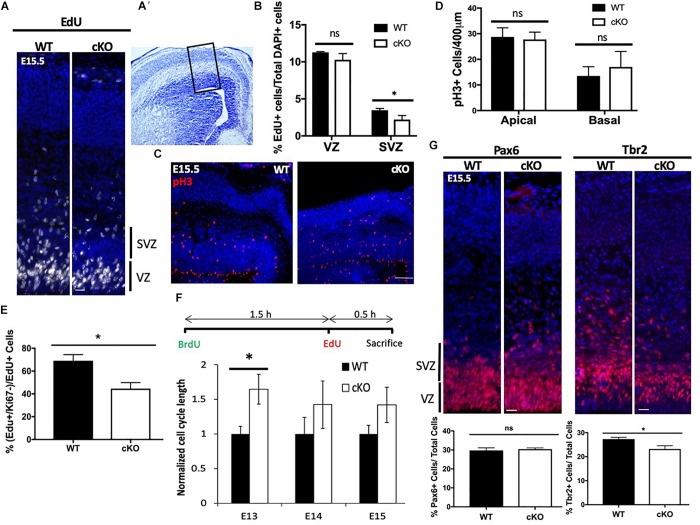
Altered cell cycle kinetics within Snf2h cKO neural progenitors. **(A)** Representative immunofluorescent images of DAPI stained nuclei (blue) and 2 h. EdU pulse-labeled cells (white) at E15.5. **(A’)** Cresyl violet stained coronal section indicating the region shown in the fluorescent images. The black box highlights the regions used for quantitation. **(B)** Quantitation of the proportion of EdU+ cells within the VZ and SVZ at E15.5 following a 2 h. pulse. **(C)** Representative images of phospho-Histone H3^+^ (pH3) mitotic cells (red) within the E15.5 neocortex of WT and *Snf2h* cKO mice. Sections were counterstained with DAPI (blue). **(D)** Quantitation of the proportion of pH3^+^ cells at the apical surface or located basally at E15.5. **(E)** EdU and Ki67 double-labeling experiments were quantitated to determine the proportion of cells that exited the cell cycle 24 h later (EdU^+^ and Ki67^–^ cells/EdU^+^ cells). **(F)** BrdU and EdU double-labeling experiments were used to estimate cell cycle length. Schematic shows the timing of BrdU and EdU injections. The mathematical formulas used to determine cell cycle length are provided in the Section “Materials and Methods.” Cell cycle length was normalized to WT cell cycle length and compared at three time points: E13, E14, and E15. **(G)** Representative images of RG (Pax6+) and IPCs (Tbr2+) immunostaining at E15.5 in WT and *Snf2h* cKO neocortex. Quantitation shown below the images indicates a reduction in IPCs but not in RG in mutant samples relative to controls. Values in all graphs are presented as the mean ± SEM; *n* = 4 mice per genotype; Student’s *t*-test, ^∗^*P* < 0.05, ns = not significant. Scale bars **(A,G)** 20 μm; **(C)** 50 μm. *P* < 0.05, ns, not significant. Scale bar in **(A,C,G)** are 20, 50, and 20 μm, respectively.

Indeed, loss of other chromatin remodeling proteins in the developing neocortex have been shown to impair progenitor growth including ATRX and the SWI/SNF proteins (Berube et al., 2005; Tuoc et al., 2013). As such, we asked whether the loss of Snf2h impaired progenitor proliferation or cell cycle kinetics. Time-mated dams (E15.5) were injected with EdU and pups were harvested 2 h later to examine the proportion of cells in S-phase. We observed a significant decrease in the number of EdU positive cells in the SVZ of *Snf2h* cKO mice compared to controls, and the VZ was not significantly different (Figures 2A,B). We also analyzed the proportion of mitotic cells dividing apically or basally using antibodies to phosphorylated-histone H3 (PH3) but did not observe any differences in the number of PH3^+^ cells in the mutants in either region (Figures 2C,D). Taken together, this suggests that the VZ of the *Snf2h* cKO mice contains a similar proportion of progenitors in S- and M-phase as the WT mice but the IPCs within the SVZ appear to have a cell cycle kinetic defect as we observed fewer S-phase cells in this region.

We next examined the proportion of cells that exited the cell cycle by injecting EdU at E14.5 then harvesting the brains 24 h later for co-immunolabeling with the cell cycle marker Ki67. Double positive cells are indicative of progenitors that have remained in the cell cycle, while EdU^+^ and Ki67^–^ cells represent those that have exited the cell cycle. We observed a reduced number of EdU^+^ and Ki67^–^ cells in the *Snf2h* cKO VZ and SVZ compared to their littermate controls (Figure 2E), suggesting that the mutant progenitor cells are either taking longer to progress through the cell cycle, or are lost by cell death. To further evaluate if the mutant progenitor cells have a prolonged cell cycle length we performed an EdU and BrdU double labeling assay that is used to calculate S-phase time and extrapolate cell cycle length (Martynoga et al., 2005; Yip et al., 2012). We performed this assay at E13.5, E14.5, and E15.5 determining that the *Snf2h* cKO progenitor cell cycle length at E13.5 was significantly longer than control progenitors (Figure 2F). At later times the *Snf2h* cKO progenitors continued to have a prolonged cell cycle length although the differences were smaller and did not reach significance (Figure 2F). Taken together, these experiments indicate that the *Snf2h* cKO neural progenitors have prolonged cell cycle kinetics and produce fewer neurons thereby contributing to the reduced size of the brain.

The RGCs can divide asymmetrically to produce a neuron and an IPC which can further divide 1–3 more times, thereby increasing neuronal output (Adnani et al., 2018). Since we observed a reduced neuronal output (decreased cell cycle exit) and fewer EdU^+^ cells located in the SVZ, we reasoned that the IPC production and/or proliferation might be compromised. The transition from RG in the VZ to IPCs in the SVZ is associated with the upregulation of Tbr2, a T-domain transcription factor, and downregulation of Pax6 (Englund et al., 2005). Thus, we next immunolabeled *Snf2h* cKO and control littermates with Tbr2 and Pax6 at E15.5. We did not observe any differences in the number of Pax6^+^ RG in the VZ but did observe a reduction in the number of Tbr2^+^ IPCs in the SVZ, consistent with a reduction in IPCs but not RG cells (Figure 2G). The reduced number of Tbr2^+^ cells suggests that the IPCs may be most susceptible to loss of Snf2h. Moreover, these results are in stark contrast to *Smarca1* targeted mice where we observed larger brains and an increased number of IPCs (Yip et al., 2012). Taken together, these results suggest that Snf2h is critical for the proliferation of Tbr2^+^ IPCs that give rise to differentiating FoxG1^+^ subtypes of the CP (Miyoshi and Fishell, 2012; Kumamoto et al., 2013).

### Snf2h Ablation Results in Increased Cell Death During IPC Expansion

**FIGURE 3:**
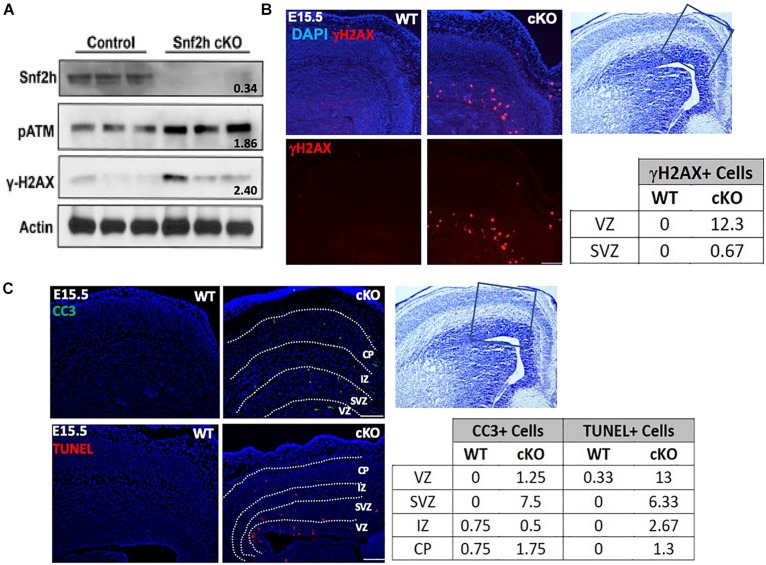
Cell loss is associated with an activated DNA damage response. **(A)** Immunoblots from E15.5 cortical extracts demonstrate increased levels of phosphorylated-ATM (pATM) protein and γ-H2AX in *Snf2h* cKO mice. Actin was used as loading control. Values denote averaged pixel densitometry normalized to Actin and relative to control samples, *n* = 3 mice per genotype. **(B)** Representative images in the developing neocortex from mutant and control mice of γ-H2AX^+^ cells (red) at E15.5. Sections were counterstained with DAPI (blue). Table shows the average number of γ-H2AX^+^ cells within the VZ and SVZ (*n* = 3 mice per genotype). **(C)** Representative images in the developing neocortex from mutant and control mice of cleaved caspase 3^+^ (CC3; top) and TUNEL^+^ (bottom) cells at E15.5. Dotted lines demarcate VZ (ventricular zone), SVZ (subventricular zone), IZ (intermediate zone), and CP (cortical plate). Cresyl violet stained section on the right panel shows the approximate location of epifluorescent images. The average number of CC3^+^ and TUNEL^+^ cells for each cortical region is shown in the table (bottom right; *n* = 3 mice per genotype). Scale bars, 100 μm.

Snf2h-containing complexes WICH and ACF have been shown to facilitate replication through heterochromatin (Bozhenok et al., 2002; Collins et al., 2002; Poot et al., 2004), suggesting that the Tbr2+ progenitor cells may be prone to replication stress and subsequent mitotic catastrophe. Other studies have shown that Snf2h-containing complexes play important roles in the DNA damage response and early G2/M damage checkpoint (Aydin et al., 2014). ATM/ATR-related kinases are activated in response to replication stress caused by stalled or collapsed DNA replication forks (Awasthi et al., 2015). To further investigate whether the DNA damage response (DDR) was activated and resulted in the loss of IPCs, we assessed ATM/ATR signaling in the developing neocortex of *Snf2h* cKO and control embryos at E15.5. Cortical extracts from the mutant embryos showed a 1.86-fold upregulation of activated (i.e., phosphorylated) pATM at E15.5 relative to control samples (Figure 3A). A second early marker of DDR activation is γ–H2AX loading at sites of DNA damage (Georgoulis et al., 2017). We observed a similar upregulation of γ–H2AX by immunoblot (Figure 3A) and in the number of γ–H2AX^+^ cells in the VZ of mutant embryos (Figure 3B). The increased number of γ–H2AX^+^ cells was present as early as ∼E13.5 (Supplementary Figure 4A), and most pronounced at E15.5 (Figure 3B). Quantification of the γ–H2AX^+^ cells at E15.5 showed that they were largely located within the VZ with an average of 12 cells/500 μm of apical surface, compared to the SVZ which contained an average of 0.67 cells over the same distance (Figure 3B). To further visualize whether upregulation of the DNA-damage response resulted in cells destined for cell death, we performed TUNEL labeling and immunolabeled for cleaved caspase 3 (CC3+) in wild type and mutant embryonic cortices at E13.5 (Supplementary Figure 4B) and E15.5 (Figure 3C). TUNEL+ and CC3+ cells were readily detectable in the mutant cortices compared to the control samples where such cells were rare. We quantified the number of cells in the VZ, SVZ, IZ, and CP over 500 μm which indicated that TUNEL+ cells were prevalent in the VZ followed by the SVZ, while the CC3+ cells showed the greatest frequency in the SVZ followed by the VZ (Figure 3C). Since the TUNEL+ cells will label all double strand breaks and they showed a similar distribution to the γ–H2AX^+^ cells, we reasoned that there is significant DNA damage occurring in cells located in the VZ and increased apoptotic cells (CC3^+^) in the SVZ. From this data, we conclude that the loss of Snf2h increased replication stress in RG progenitors that subsequently compromised the generation and proliferation of IPCs, ultimately causing impaired neuronal output.

### Snf2h Loss Has the Greatest Effect on Upper Layer Neuron Output

**FIGURE 4:**
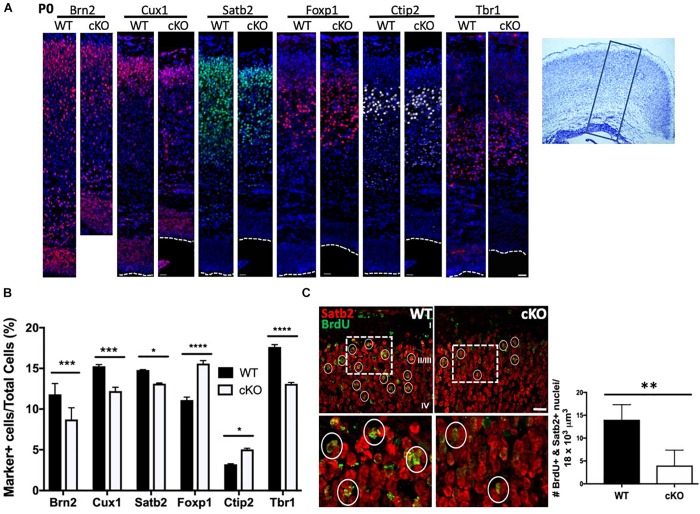
Reduced production of upper layer cortical neurons in Snf2h cKO mice. **(A)** Cortical sections from P0 brains were immunostained with antibodies to UL (layers II-IV, Brn2, Cux1, Satb2, and Foxp1) and DL (layers V-VI, Ctip2 and Tbr1) neurons, as indicated. Dotted lines indicate the position of the lateral ventricle. Box on cresyl violet stained section (rightmost panel) indicates the approximate position of the images shown on the left panels. Scale bar, 20 μm. **(B)** Cortical marker positive cells were plotted as a percentage of the total number of DAPI^+^ cells contained within a box of fixed width that contained all cells starting from the pial surface to the lateral ventricle. Values are presented as the mean ± SEM. ^∗^*P* < 0.05, ^∗∗∗^*P* < 0.01, ^****^P < 0.001, Student’s *t*-test, *n* = 4–6 mice per genotype. **(C)** UL neurons were birthdated with a single injection of BrdU at E15.5 and harvested at birth for co-immunostaining with Satb2 (red) and BrdU (green). Dotted boxes indicate the position of the higher magnification images shown below. Circles denote BrdU^+^ nuclei. Graph depicts quantitation of BrdU^+^ (>75% of nucleus stained for BrdU) and Satb2^+^ neurons from E15.5 mutant and control embryos. Five cubic bins were scored and averaged per embryo. Values are presented as the mean ± SEM. ^∗∗^*P* < 0.05, Student’s *t*-test, *n* = 3 mice per genotype.

The cortical plate is established through successive rounds of neurogenesis that further resolves into the six cortical layers of the mammalian brain (Kwan et al., 2012). Most neurons of the same laminar location share a common date of birth. For example, Layer I (Cajal-Retzius) neurons are born around E11, while layer V and VI (corticofugal and corticothalamic projection; early born or deep layer) neurons are produced between E12.5 and E14.5 (Molyneaux et al., 2007). Subsequently, layer II-IV (callosal projection; late-born or upper layer) neurons are produced from ∼E14.5 to ∼E16.5 (Fame et al., 2011). Given that we observed a slight reduction in DL neuron markers and a greater reduction in UL markers by immunoblot at E15.5 (Figure 1D), we next assessed how the reduced IPC proliferation affected the generation of postmitotic projection neurons in *Snf2h* cKO neonates. Initially we harvested and stained P0 cortices for DL and UL markers. Consistent with immunoblots at E15.5, we observed a reduced proportion of cells expressing the layer II-III markers Brn2, Cux1, and Satb2 (Figures 4A,B). Surprisingly, we observed an increased proportion of layer IV Foxp1+ cells in the mutant animals compared to controls (Figures 4A,B). Within deep layers (V and VI), we observed an increased proportion of layer V Ctip2+ cells and a reduced percentage of layer VI Tbr1+ cells (Figures 4A,B). While the Ctip2^+^ cell number does not match with what we observed on E15.5 immunoblots, it is known that alternate corticofugal identities with layers V/VI are characterized by Ctip2 and Tbr1 levels. As such, these differences could be the result of a fate change between E15.5 and birth when the layer markers were re-analyzed (McKenna et al., 2011). Regardless, the combined number of layer IV-VI marker positive cells was equivalent in WT and mutant animals suggesting that Snf2h loss does not significantly affect the generation of DL neurons but appears to influence specific cell fates within these layers. Alternatively, the overall production of marker-positive UL neurons was reduced and may be a consequence of IPC cell loss.

To further confirm that late-born neuron production was compromised we birth-dated cortical cells with a single BrdU injection at E15.5 and analyzed pups at birth. We co-labeled with Satb2 and BrdU to identify birth-dated callosal projection neurons (CaPNs; layers II-IV). In this way, we quantitated the number of brightly fluorescent BrdU-labeled cells (BrdU staining > 75% of nucleus) that co-localized with the layer marker. We found a robust decrease in the total number of BrdU^+^ and Satb2^+^ late-born neurons in the mutant cortex relative to controls (Figure 4C). Collectively, these results demonstrate an overall reduction in most cortical layers upon *Smarca5* loss with the greatest decrease observed in the upper layers.

### Snf2h Depletion Results in Altered Neuronal Projections

**FIGURE 5:**
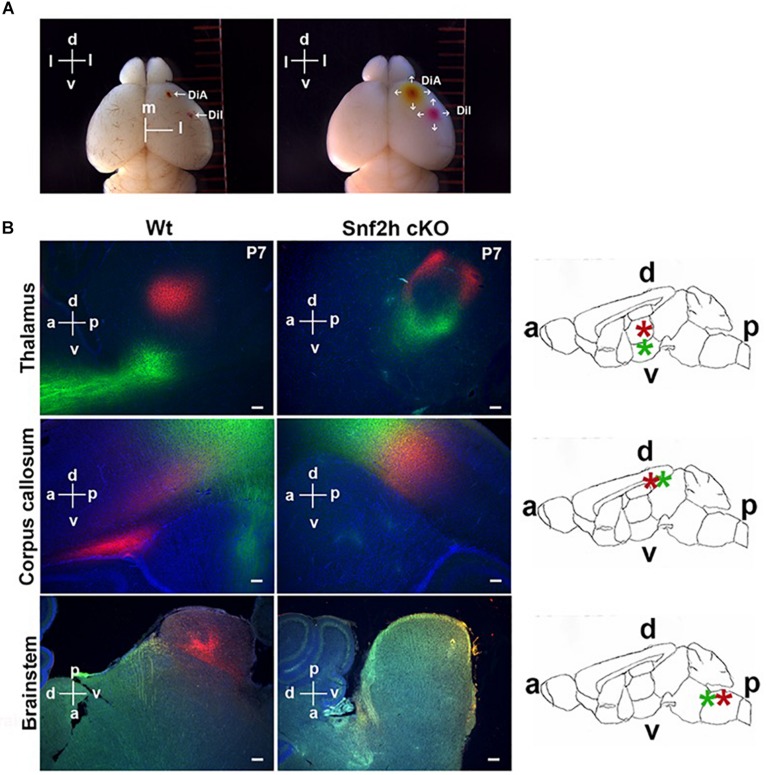
Mapping cortical projection output in Snf2h cKO. **(A)** Insertion of lipophilic tracers and diffusion throughout the brain. The left panel shows a wild type mouse brain 1 h after insertion in the pia mater of a single DiA crystal in the right motor cortex area and a single Dil crystal in the right somatosensory cortex area. The right panel shows the same brain 1 week after diffusion of both neuronal tracers. The dorsoventral (d and v) and mediolateral (m and l) orientations are indicated. **(B)** Representative epifluorescent images of both lipophilic tracers in P7 brains after 4 weeks of incubation. Dil^+^ (red) synaptic terminals were visualized within control brains in the thalamus, CC, and brainstem (left panels), but these were reduced or almost absent in the mutant brains (right panels). DiA^+^ (green) projections toward the hypothalamus (top panels) and the projection output from the cerebral cortex (middle panels) were also reduced within the mutant brain. Rightmost panels: Relative orientation of the dorsoventral (d and v) and the anteroposterior (a and p) brain. A schematic representation of the whole brain in a sagittal section is shown to highlight the corpus callosum (CC), thalamus (Th), and caudate putamen (Cp). Scale bars, 50 μm.

CaPNs are a diverse subtype of upper layer projection neurons that connect the hemispheres of the cerebral cortex through the corpus callosum and play multiple roles in cognition (Fame et al., 2011). The corticothalamic neurons receive inputs from sensory neurons and relays it to the thalamus (Grant et al., 2012). We first visualized the trajectory of cortical neurons in P7 brains by co-insertion of lipophilic crystal dyes into the motor (DiA; green) and somatosensory cortex areas (Dil; red) that were incubated in darkness for a period of 4 weeks before analysis. DiA+ (green) output projections from the cerebral cortex to the thalamus and alongside the corpus callosum (CC) were absent in the Snf2h cKO brains (Figure 5B, top and middle panels). At this age, we also observed an intense accumulation of Dil+ (red) terminal projections forming at one extremity of the normal CC (Figure 5B, middle panel). In the mutants, the CC is poorly developed at P7 and we did not observe the concentrated zone of Dil+ (red) accumulation that was present in the control animals. Moreover, the DiI+ synaptic terminals in the thalamus were reduced (Figure 5B, top panel) and there was a dramatic reduction in projections to the brainstem (Figure 5B, bottom) of mutant mice.

We also analyzed P21 brains from WT and *Snf2h* cKO mice at 6 weeks post-insertion (Dil and DiA). Similar to our observations in younger mice, there was a severe reduction in Dil+ (red) terminals within the thalamus of mutant mice (Supplementary Figures 5A,A’) and also in their CC (Supplementary Figures 5B,B’) relative to controls. DiA+ (green) projections through the CC were almost absent in more affected animals (Supplementary Figure 5B”). Interestingly, aberrant DiA+ (green) neuronal cortical projections were observed in the caudate putamen and in the basal forebrain of mutant mice (Supplementary Figures 5C,C’). Taken together, the dye tracing experiments indicate that corticothalamic neurons and neurons that project across the CC both show altered neuronal projections from the cortical layers in the *Snf2h* cKO mice. In addition, we observed mutant projections to novel regions suggesting that some axonal projections are mistargeted, as well as poorly developed.

**FIGURE 6:**
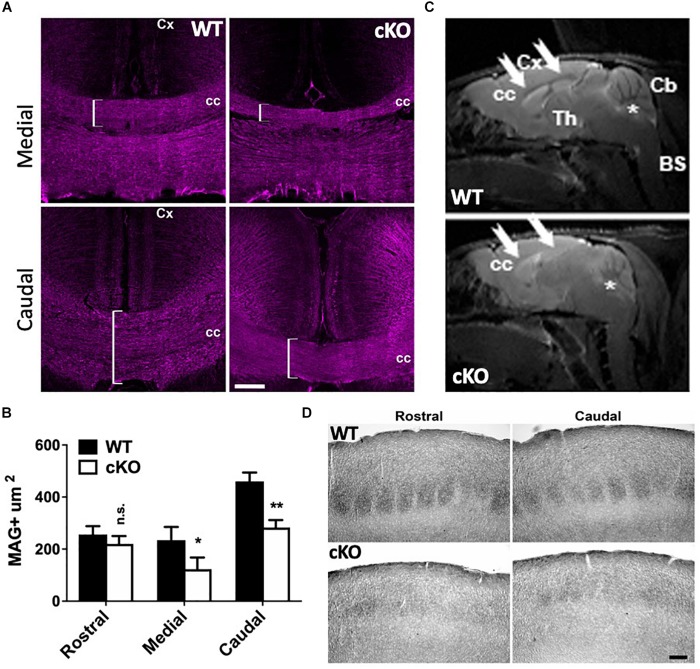
Corpus callosum dysgenesis and disrupted targeting of callosal projection neurons. **(A)** P40 medial and caudal coronal sections through the CC of mutant and control mice immunolabeled for myelin-associated glycoprotein (MAG; magenta), a marker of myelinated axonal fibers. Note the reduction of MAG^+^ fibers in the medial and caudal mutant brain (brackets). Cc, corpus callosum; Cx, cortex. Scale bar, 100 μm. **(B)** Quantitation of MAG+ areas through the corpus callosum at P40. 5 cubic bins were scored and averaged per section. Values are presented as the mean ± SEM. ^∗^*P* < 0.05, ^∗∗^*P* < 0.01, Student’s *t*-test, *n* = 3–4 sections per region per genotype. **(C)** Magnetic resonance imaging (MRI) of *Snf2h* cKO-Emx1 and control mice at P180 through the sagittal plane. Note the reduced white matter content (seen as gray) through the CC (arrows) in mutant mice relative to controls. Asterisks denote the cerebellum. CC, corpus callosum; Cx, cortex; Th, thalamus; Cb, cerebellum; BS, brain stem. 3 animals per genotype were scanned in 4 dimensional planes. **(D)** Brightfield images from P40 brains showing cytochrome oxidase staining through the somatosensory cortex. Note the altered barrel structures in the mutant cortex relative to controls. Scale bar, 100 μm.

Since the lipophilic dye labeling suggested reduced projections through the CC, we further investigated its development. Adult coronal sections through rostral, medial and caudal regions from mutant and control brains were immunolabeled with myelin-associated glycoprotein (MAG), a cell membrane glycoprotein expressed in myelinated axons that connect the contralateral hemispheres. We did not detect significant differences in the area of MAG^+^ axons through the rostral brain despite an obvious trend for decreased size (Supplementary Figure 6). However, there was a significant reduction in the area covered by MAG^+^ axons in the medial and caudal regions of the mutant CC relative to controls (Figures 6A,B). We also observed these abnormalities using magnetic resonance imaging (MRI). The CC is severely reduced in mutant brains relative to controls in adult mice (Figure 6C, white arrows), suggesting that the loss of CaPNs during embryonic development results in dysgenesis of the CC.

To further corroborate the defect in corticothalamic projections we examined whether there were abnormalities in the topography maps. The somatosensory cortex is structured such that input from individual whiskers is organized into discrete barrel structures that can be identified after staining with cytochrome oxidase (Wong-Riley and Welt, 1980). Rostral and caudal sections from P40 control mice show organized barrel structures whereas *Snf2h* cKO mice lacked the densely and uniformly stained barrel structures (Figure 6D). Taken together, forebrain ablation of the *Smarca5* gene resulted in reduced production of cortical neurons and axonal targeting defects to the thalamus and through the CC to the contralateral side.

### Snf2h cKO Mice Are Hyperactive

**FIGURE 7:**
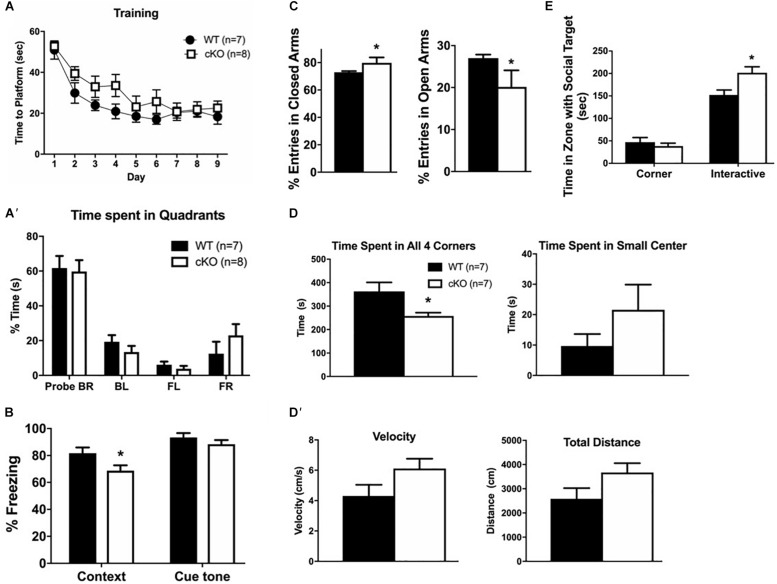
Cortical projection neuron dysgenesis alters cognitive function. **(A)** Morris water maze training sessions. The time it took the mice to reach the platform was recorded. No statistically significant differences were detected between genotypes during 9 days of training. **(A’)** Trial day 10 in the Morris water maze reveals no differences in the percentage of time spent in the different quadrants. Both WT and mutant mice spent most time in the BR quadrant that contained the platform. **(B)** The fear-conditioning assay reveals that mutant mice responded normally to the cue tone after training but have a reduced context-dependent response relative to controls. **(C)** The elevated plus maze reveals that mutant mice have a higher percentage of total entries into the closed arms, but a lower percentage of entries into the open arms relative to controls. **(D)** The open field test reveals that mutant mice spent more time in the brightly lit center and less time in any of the corners. However, this was not a function of speed or total distance traveled **(D’)** as they showed no difference to control animals. **(E)** The social interaction assay reveals that mutant mice spent more time interacting with a stranger mouse than with an inanimate object relative to controls. For all tests, *n* = 7–10 mice per genotype were used, values are presented as the mean ± SEM. ^∗^*P* < 0.05, one-way ANOVA, except for panels **(A’,E)**, which utilized two-way ANOVA with multiple comparisons.

To assess whether the reduced number and altered organization of cortical projection neurons resulted in cognitive deficits, we performed a battery of neurobehavioral tests in adult mutant and control littermates to assess learning and memory (Morris water maze, fear conditioning), and anxiety-related symptoms (elevated plus maze, social interactions, open field). The Morris water maze assay showed no differences between mutant and control littermates in the time needed to reach a platform during 9 days of training (Figure 7A); and no differences in the time spent in all quadrants at day 10 after training (Figure 7A’). In the fear conditioning assay, mice are exposed to a novel cue (“cue tone”), followed by a foot-shock (“context”), and the “fear” response is measured (“conditional response”). *Snf2h* cKO mice had a normal response to the cue tone compared to controls, but a reduced response to the context, suggestive of impaired associative learning skills (Figure 7B). In the elevated plus maze, an assay of anxiety, mutant mice showed an increased percentage of total entries into the closed arms, and lower entries into the open arms relative to control littermates (Figure 7C). While this test suggested increased anxiety-like behavior, the social interaction assay demonstrated decreased anxiety-like behavior as the *Snf2h* cKO mice spent more time interacting with a stranger mouse compared to control littermates (Figure 7E). We also assessed mice in the open field arena, a third assay of anxiety. *Snf2h* cKO mice displayed a significant decrease in the time spent in all 4 corners relative to control littermates again suggesting that they have reduced anxiety-like behavior (Figures 7D,D’). Taken together, the reduced cortical projection output along with altered topography and dysgenesis of the CC contributes to impaired associative learning and alterations in anxiety-like behaviors in the *Snf2h* cKO mouse model.

### Snf2h Activates the Protocadherin Gene Cluster

**FIGURE 8:**
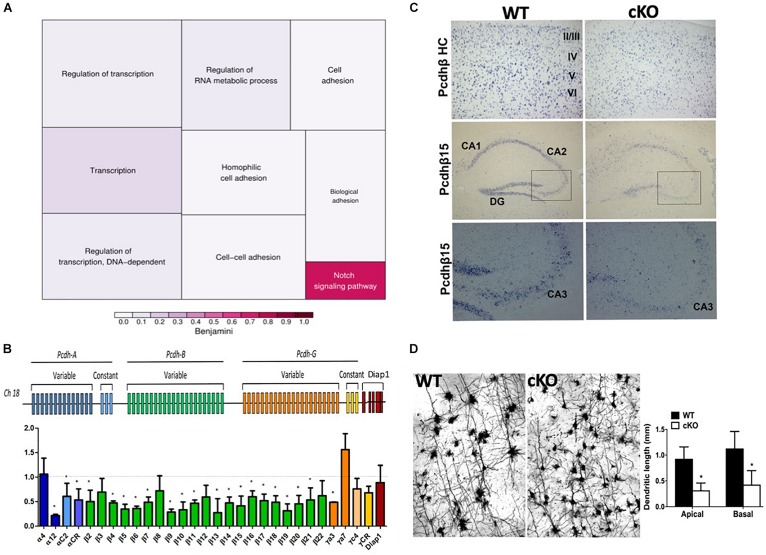
Altered expression of the protocadherin gene cluster. **(A)** Gene ontology (GO) of differentially expressed transcripts from P0 mutant vs. control cortical extracts hybridized to mouse genome Affymetrix Gene 1.0 ST microarrays. Also refer to Table 1. **(B)** Top: Schematic diagram of the clustered protocadherin genes. Bottom: RT-qPCR fold-change expression changes in the mutant cortex relative to control littermates in the P30 cortex. Triplicate samples were averaged from 3 independent cortical preparations per genotype and normalized to L32 expression. ^∗^*P* < 0.05, Student’s *t*-test. **(C)** *In situ* hybridization for the *Pcdh-ß* heavy chain (HC; labels all *Pcdh-ß* genes) and *the Pcdh-ß15* gene through the mutant and control cortex and hippocampus at P100. Boxed regions in middle panels are shown in the bottom panels. Note the reduced hybridization signal within the nuclei of cortical and hippocampal neurons in the mutant cortex relative to controls. **(D)** Z-stacks of Golgi-Cox stained cortical projection neurons through the *Snf2h* cKO and control cortex at P200. Note the abnormal arborization of mutant neurons. Graph depicts the dendritic length measurements from apical and basal dendrites. Values are presented as the mean ± SEM. ^∗^*P* < 0.05, Student’s *t*-test, *n* = 3-4 sections per genotype.

ISWI is bound at the transcriptional start site of many genes whereit remodels nucleosomes to establish/maintain a nucleosome free region (Sala et al., 2011). Indeed, Snf2h loss has a significant impact on neuronal expression programs in the developing cerebellum (Alvarez-Saavedra et al., 2014). As such, we postulated that altered target gene transcription might underlie the cognitive deficits by altering the formation and function of neuronal networks. In mice, synaptogenesis commences at birth, hence we isolated RNA extracts from mutant and control cortices at P0 and hybridized them to mouse genome Affymetrix Gene 1.0 ST microarrays. After a stringent filtering analysis (>1.5-fold change, *P* < 0.01), we identified 45 downregulated and 20 upregulated genes in the *Smarca5*-null neocortex relative to controls (*n* = 2 mice per genotype, Table 1). Gene ontology (GO) analysis revealed enrichment for genes involved in cell adhesion and transcriptional control (Figure 8A and Table 1). Of specific interest, six different isoforms of the *Protocadherin-ß (Pcdh-ß*) gene cluster were downregulated in the mutant cortex (Table 1). The protocadherin cluster controls the largest subclass of the cadherin-repeat containing cell adhesion family of proteins with the majority of the *Pcdh* genes residing in three adjacent gene clusters, designated α (*Pcdh*-α*), ß (Pcdh- ß)*, and γ *(Pcdh-*γ) (Figure 8B). Stochastic and combinatorial expression of the *Pcdh-*α, *-ß*, and *-*γ protein isoforms allows for increased diversity through the formation of *cis*-tetramers within a neuron, which then engage in homophilic interactions to specify neuronal connections (Hirayama et al., 2012; Thu et al., 2014; Goodman et al., 2016; Mountoufaris et al., 2017, 2018). Thus, we analyzed the expression of multiple clustered *Pcdh-*α, -*ß*, and *-*γ isoforms by RT-qPCR from the adult cortex. Analysis of P30 cortical extracts revealed that the mutant neocortex displayed altered expression of almost all the *Pcdh-ß* isoforms, as well as several *Pcdh-*α *and Pcdh-*γ isoforms compared to control littermates (Figure 8B). Similarly, we observed reduced expression of *Pcdh-ß* by *in situ* hybridization throughout the adult cortex using a probe that recognizes all *ß* isoforms (Figure 8C, top panels). Moreover, a probe directed exclusively against the *Pcdh-ß15* isoform also exhibits reduced expression levels within the hippocampus by *in situ* hybridization (Figure 8C, middle and bottom panels). These results suggest that Snf2h is necessary for the activation and expression maintenance of the clustered *Pcdh-ß* isoforms within the mammalian cortex.

**TABLE 1:** List of altered genes from the Snf2h cKO-Emx1 cortex.

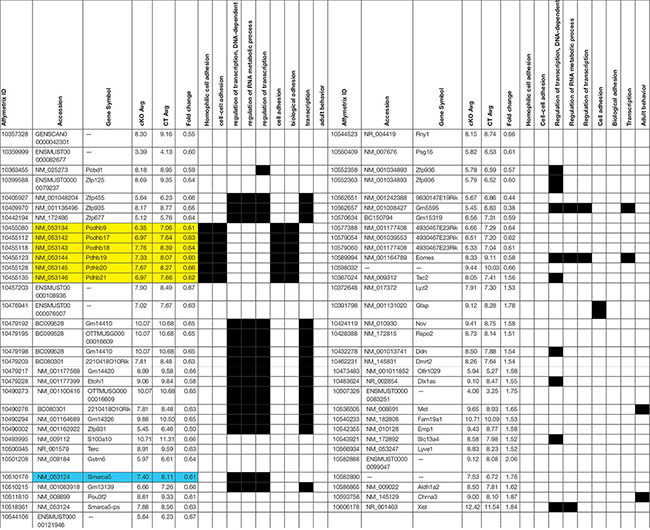

*Two microarrays per genotype were averaged from mutant and control cortices obtained at P0. Each sample per genotype is a pool of 3 cortices from individual pups. Deregulated genes denote *P* < 0.01. Differentially expressed genes were analyzed by Significance Analysis of Microarrays (SAM). Yellow highlights fold-change for *Pcdh-ß* genes and blue highlights fold-change for *Snf2h (Smarca5).**

Previous studies have demonstrated that aberrant *Pcdh* isoform expression results in severely reduced neuronal arborization and altered dendritic self-avoidance (Wang et al., 2002; Weiner et al., 2005; Lefebvre et al., 2012; Ledderose et al., 2013). Interestingly, other studies found that Satb2-deficient neurons clump together and show poor dendritic expansion, which was attributed to an imbalance in the adhesive properties of the neurons (Zhang et al., 2012). As such, we assessed the functional significance of clustered *Pcdh* gene deregulation in *Snf2h-*null cortical neurons with Golgi-Cox staining. Imaging stained sections revealed the atrophied arborization and reduced basal and apical dendrites of cortical projection neurons within the adult mutant cortex relative to controls (Figure 8D). We conclude from these experiments that Snf2h is necessary for the proper activation of the clustered *Pcdh* genes to mediate postmitotic dendritic arborization.

## Discussion

The development of the cerebral cortex is dependent on the balance between stem cell maintenance and TF-mediated differentiation to produce neuronal subtypes in the proper order and correct number. Recent work has demonstrated that ISWI chromatin remodeling is critical to this process. We previously demonstrated that *Smarca1* deletion and subsequent loss of Snf2l remodeling activity resulted in FoxG1 upregulation, hyperproliferation and delayed differentiation of cortical progenitors (Yip et al., 2012). FoxG1 is necessary for the appropriate spatiotemporal expansion, differentiation and organization of nearly all cortical subtypes, early postnatal development of hippocampal neurons, and axon trajectory of CaPNs (Miyoshi and Fishell, 2012; Tian et al., 2012; Kumamoto et al., 2013; Cargnin et al., 2018). Here we report that Snf2h is also necessary for proper FoxG1 activation and neocortical expansion. Snf2h loss following conditional deletion of the *Smarca5* gene resulted in increased cell death and a lower production of Tbr2^+^ IPCs and FoxG1^+^ post-mitotic neurons in the cortical plate by mid-neurogenesis. In addition, we demonstrate that Snf2h loss results in a dramatic reduction in the specification of multiple UL neuron subtypes. The poor production of Tbr2^+^ IPCs resulted in fewer UL neurons but did not significantly compromise DL neuron production. It should be noted that there is also a potential fate change in the deep layers (increased Ctip2^+^ cells; decreased Tbr1^+^ cells) that was similar to our findings with the *Smarca1* deleted mice and will require additional work to characterize at the molecular level (Yip et al., 2012).

Previous studies have indicated that increased DNA replication stress and DNA damage in cortical progenitors can deplete the progenitor pool, ultimately resulting in fewer late born neurons that reside in the upper layers (Tuoc et al., 2013; Huh et al., 2016; Nitarska et al., 2016; Chen et al., 2017). This is certainly a strong possibility to explain some of the phenotype observed in the *Snf2h* cKO mice. ISWI chromatin remodeling has been implicated in double strand DNA break (DSB) repair via homology-directed recombination and non-homologous end-joining mechanisms, and single stranded breaks using base excision repair or transcription coupled nucleosome excision repair mechanisms (Aydin et al., 2014). Studies with Atrx knockout mice have shown that replication through heterochromatin is compromised leading to DSBs and genomic instability (Huh et al., 2012, 2016). A similar mechanism could be at play in the *Snf2h* cKO mice since previous studies have shown a requirement for both the ACF (ACF1 and Snf2h) and WICH (WSTF and Snf2h) complexes in proper replication of heterochromatin, the latter complex via a direct interaction with the PCNA protein (Collins et al., 2002; Poot et al., 2004; Torigoe et al., 2011). Moreover, Snf2h was identified in a large screen for proteins present at stalled and collapsed replication forks (Sirbu et al., 2013). In this regard, we observed increased cell cycle length and γH2AX+ cells in the VZ of *Snf2h* cKO mice compared to control animals suggestive of increased replication stress (Figures 2, 3). Improper repair of the DSBs can result in mitotic catastrophe in progenitors that continue to divide, as shown in the Atrx mutant mice (Huh et al., 2016). Consistent with this model, the *Snf2h* cKO mice show a reduced number of proliferating Tbr2+ intermediate progenitors and an increase in cleaved caspase 3 staining within the SVZ (Figures 2, 3). Interestingly, ACF, RSF, and WICH have all been shown to be rapidly recruited to DSBs where they promote phosphorylation and subsequent incorporation of γH2AX at sites of DNA damage (Xiao et al., 2009; Lan et al., 2010; Sheu et al., 2010; Sanchez-Molina et al., 2011; Aydin et al., 2014). Although the precise details need to be flushed out, the Snf2h complexes have also been shown to interact with Sirt6, RNF20, PARP, NuMA, or Ku proteins at DSBs thus indicating their involvement in both HDR and NHEJ repair processes (Smeenk et al., 2013; Toiber et al., 2013; Aydin et al., 2014; Min et al., 2014; Vidi et al., 2014; Atsumi et al., 2015). Regardless of the precise mechanism utilized, Snf2h ablation hampers DNA repair that could lead to a secondary reduction in the upper layer neurons. One caveat to this mechanism is that Snf2l expression remains in these animals, suggesting that it could compensate to re-start stalled replication forks and repair DSBs, although its role in DNA replication and repair is less well understood.

Our studies suggest that both Snf2h and Snf2l co-modulate *FoxG1* expression but with differential consequences, thereby establishing post-mitotic cortical identity and proper neural output to control brain size (Yip et al., 2012; Kumamoto et al., 2013). Indeed, we have observed similar results in the cerebellum, where Snf2h and Snf2l co-regulate the expression of the *Engrailed-1* gene, a master regulator of cerebellar patterning (Sillitoe et al., 2010; Alvarez-Saavedra et al., 2014). While the precise chromatin-associated mechanisms of how Snf2h and Snf2l antagonistically co-modulate target loci remain unknown, there are several possibilities that could contribute to the proper regulation of corticogenesis. Recent work has suggested that Snf2h and Snf2l are interchangeable within all ISWI complexes (Oppikofer et al., 2017), thus suggesting that intrinsic differences in remodeling activity between Snf2h and Snf2l coupled with their developmental expression differences could mediate subtle transcriptional changes at key homeotic TFs (like FoxG1) that subsequently affects IPC proliferation and/or differentiation.

Our work has also uncovered a role for Snf2h in the functional maturation of postmitotic cortical projection neurons. We demonstrate that Snf2h loss results in the abnormal development of neuronal projections, as we did not observe barrel structures in the somatosensory cortex, corticothalamic projections were altered, and the animals presented with CC dysgenesis. Similar findings have been observed in both *Satb2* and *FoxG1* cKO mice generated with forebrain-specific Cre driver lines (Cargnin et al., 2018; Zhang et al., 2019). Interestingly, the Satb2 cKO mice had a range of behavioral deficits that showed some overlap to our analysis of the *Snf2h* cKO mice (e.g., abnormal social novelty).

Although each of these models showed agenesis of the CC, it remains to be determined if this results from interactions between these different proteins, regulation of *Satb2* by Snf2h (and Snf2h regulation of *FoxG1*), or dysregulation of common target genes. Alternatively, the nuclear matrix-associated protein Satb2 may be mediating chromatin loop formation for Snf2h-dependent chromatin remodeling and subsequent transcriptional activation. In the Snf2h cKO mice we observed altered expression of the clustered *Pcdh* genes that could explain the arborization defects. Genetic manipulation of the *Pcdh-*α and *Pcdh-*γ clusters has suggested that these proteins mediate synapse formation and neurite self-avoidance (Takeichi, 2007; Kostadinov and Sanes, 2015). Other studies have indicated that *Pcdh-*α null mice have abnormal axon projections in olfactory and serotonergic neurons while *Pcdh-*γ cKO mice have altered branching and arborization within cortical neuron dendrites (Hasegawa et al., 2008; Katori et al., 2009; Garrett et al., 2012). While no studies have eliminated the function of the *Pcdh-ß* genes specifically, *CTCF* cKO mice have diminished cortical and hippocampal expression of all but one of the *Pcdh-ß* isoforms and 53 of the 58 genes in the clustered *Pcdh*s much like our observations with the *Snf2h* cKO mice (Hirayama et al., 2012).

Could it be that Snf2h-driven chromatin remodeling is instrumental to *Pcdh-ß* gene expression? In this regard, *Pcdh* gene expression depends on CTCF/cohesin loading that promotes the long-range DNA looping necessary for differential isoform usage (Yokota et al., 2011; Guo et al., 2012). Moreover, deletion of *CTCF*, *Nipped-B-like*, or other cohesin subunits results in cortical hypoplasia (Kawauchi et al., 2009; Cuadrado et al., 2012; Remeseiro et al., 2012). While the precise function of *Snf2h* in *Pcdh* gene expression will require further investigation, Snf2h was shown to interact directly with Rad21, a subunit of the cohesin complex (Hakimi et al., 2002). A Snf2h interaction with Satb1 has also been shown to mediate nucleosome positioning over long-range chromatin domains (Yasui et al., 2002). Given that Satb2 is closely related to Satb1, a Satb2/Snf2h interaction could be involved in the chromatin looping regulation of the clustered *Pcdh* genes.

Human mutations in both FOXG1 and SATB2 result in intellectual disability disorders (Kortum et al., 2011; Florian et al., 2012; Usui et al., 2013; Zarate et al., 2017). Interestingly, the *Satb2* cKO mice had a range of behavioral deficits that showed some overlap to our analysis of the *Snf2h* cKO mice, leaving one to surmise that *Smarca5* should also be considered as a candidate gene or genetic modifier for intellectual disability disorders with CaPN defects and CC dysgenesis.

## Data Availability Statement

All datasets generated for this study are included in the manuscript/Supplementary Files.

## Ethics Statement

All animal experiments were approved by the University of Ottawa’s Animal Care ethics committee, with the guidelines set out by the Canadian Council on Animal Care.

## Author Contributions

MA-S conceived, designed and executed all experiments with the technical support of KY, unless stated otherwise. LH performed the Pcdh RT-qPCR analyses. YD and RK performed and analyzed the DiA/DiI labeling experiments. DY and II analyzed microarrays. TH and TY performed and analyzed *in situ* hybridization experiments. NC and SS performed immunoblots and EdU/BrdU co-labeling experiments on the embryonic cortex, respectively. DP supervised and provided funding for the project. MA-S and DP wrote the manuscript.

## Conflict of Interest

The authors declare that the research was conducted in the absence of any commercial or financial relationships that could be construed as a potential conflict of interest.

## Footnotes

1 rsbweb.nih.gov/ij/

2 https://imagej.nih.gov/ij/
