# Supplementary material for: Snf2h Drives Chromatin Remodeling to Prime Upper Layer Cortical Neuron Development
Source: Front Mol Neurosci. 2019 Oct 17;12:243. doi: 10.3389/fnmol.2019.00243 (PMC6811508; doi:10.3389/fnmol.2019.00243)
Supplement: Supplementary file 1 [file Data_Sheet_1.docx]

**SUPPLEMENTAL INFORMATION**

**Snf2h drives chromatin remodeling to prime upper layer cortical neuron development**

*Matías Alvarez-Saavedra^1,3,6^, Keqin Yan^1^, Yves De Repentigny^1^ Lukas E. Hashem^1^, Nidhi Chaudary^1^, Shihab Sarwar^1^, Doo Yang^2,4^, Ilya Ioshikhes^2,4^, Rashmi Kothary^1,2,3^, Teruyoshi Hirayama^5^, Takeshi Yagi^5^ and David J. Picketts^1,2,3,7^.*

**
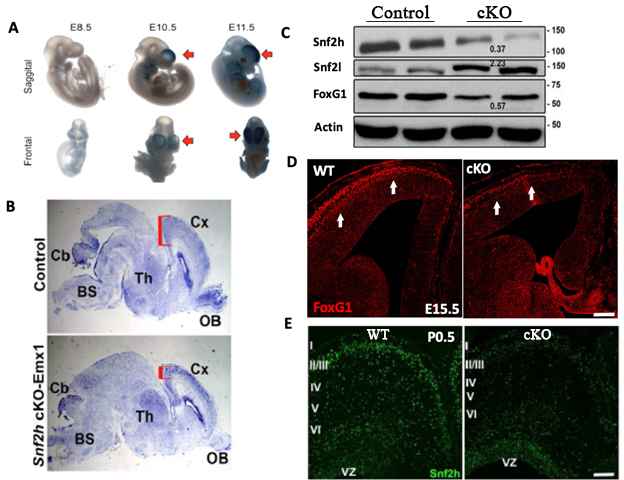
**

**Supplemental Figure 1: Phenotypic characterization of *Snf2h* cKO by Emx1-Cre brains.**

**A)** Emx1-Cre driver mice (Gorski et al., 2002) were bred to ROSA-STOP-LacZ mice that activates lacZ expression upon Cre deletion. Note the expression of LacZ (blue-colored product) predominantly in the developing telencephalon after ~E10.5 (arrows) in Emx1-Cre^-/+:^::ROSA-STOP-lacZ^-/+^ mice. **B)** Nissl staining of P1 sagittal sections from mutant and control brains Note the reduced cortical area of mutant neonates (red brackets). OB, olfactory bulb; Cx, cortex; Th, thalamus; Cb, cerebellum; BS, brain stem. **C)** Immunoblots for Snf2h, Snf2l, FoxG1 and Actin, as loading control, from mutant (cKO) and control cortices at E15.5. Values denote averaged pixel densitometry normalized to actinand relative to control samples, n=4 mice per genotype. Relative protein sizes in kDa are shown on the right. **D-E)** Confocal Z-stacks through the mutant and control neocortex immunolabeled for FoxG1 (red) at E15.5 or Snf2h (green) at birth (P0.5). Arrows highlight FoxG1+ progenitors throughout the cortical plate that are reduced in mutant cortices relative to controls. Also note the reduced number of Snf2h^+^ cells in the mutant neocortex. VZ, ventricular zone. I-VI Roman numerals refer to the cortical layers. Scale bars, D) 100μm; E) 50μm. Related to Figure 1.

**Supplemental Figure 2: *Snf2h* cKO by Emx1-Cre mice do not have alterations in cerebellar development.** Epifluorescent images of P10 (top panels) and P60 (bottom panels) sagittal sections through the CB counterstained with DAPI (blue) of *Snf2h* cKO by Nestin-Cre mice (top right panel; Alvarez-Saavedra et al, 2014) and *Snf2h* cKO by Emx1-Cre mice mice (this article; bottom right panel), relative to their corresponding control littermates. Note the robust decrease in cerebellar size in the Nestin-cKO mouse model, but not in the Emx1-cKO mouse model. Scale bar, 100μm. Related to Figure 1.

**
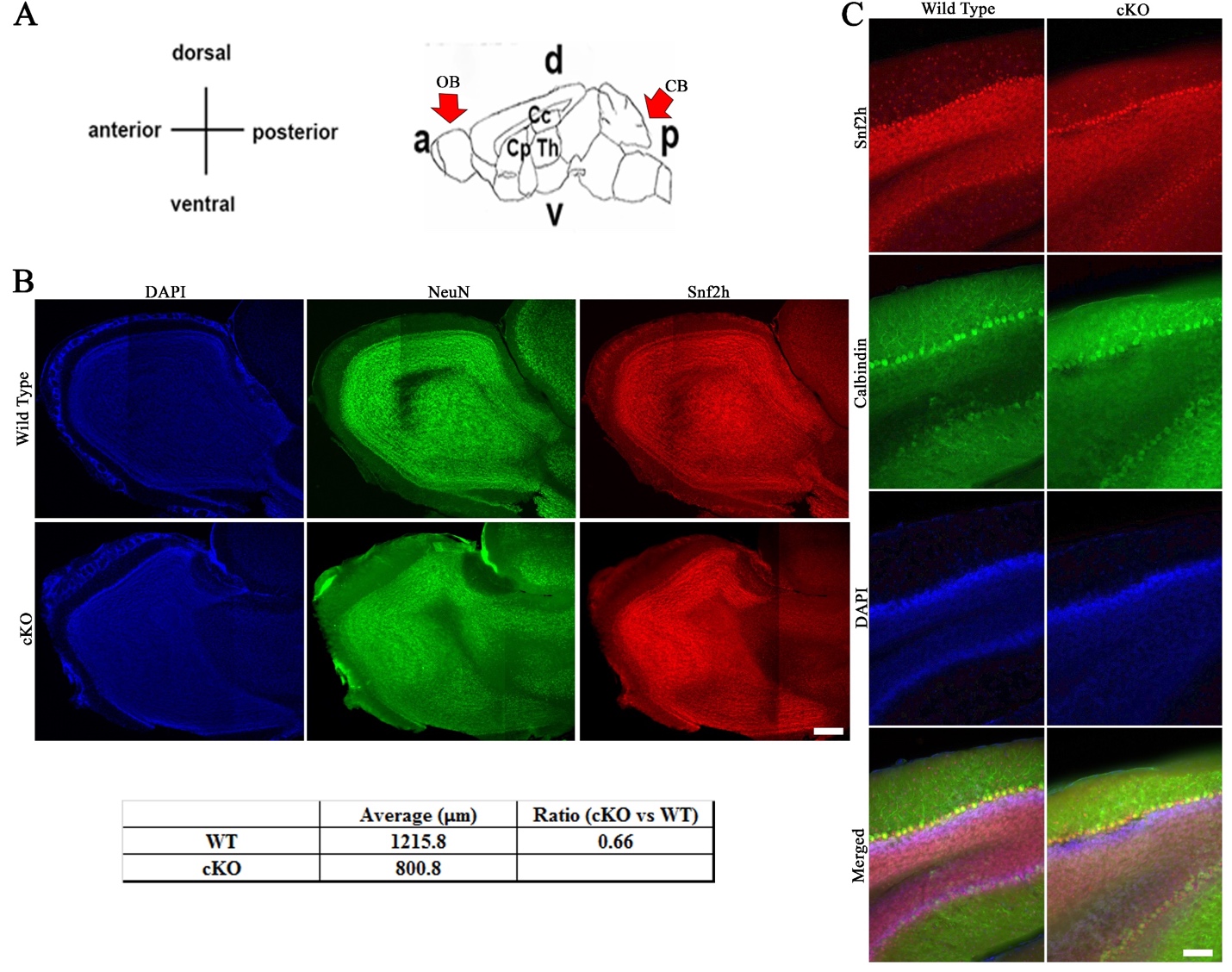
**

**Supplemental Figure 3: *Snf2h* cKO mice have a reduction in olfactory bulb size, but no alterations in cerebellar development where Snf2h is robustly expressed.**

**A)** Relative orientation of the dorsoventral (d and v) and the anteroposterior (a and p) brain. A schematic representation of the whole brain in a sagittal section is shown to highlight the corpus callosum (CC), thalamus (Th), caudate putamen (Cp), olfactory bulb (OB) and cerebellum (CB). **B)** Epifluorescent images of P60 sagittal sections through the OB showing expression of NeuN (green), a neuronal marker, Snf2h (red) and counterstained with the nuclear marker DAPI (blue). Measurements below show the mean average of cKO and control olfactory bulbs at P60. n=3 mice per genotype. **C)** Epifluorescent images of P60 sagittal sections through the CB showing expression of Calbindin (green), a Purkinke neuron marker, Snf2h (red) and counterstained with the nuclear marker DAPI (blue). Merged images are provided in the bottom panels. Note the robust expression of Snf2h in both genotypes. Scale bars, 100μm. Related to Figure 1.


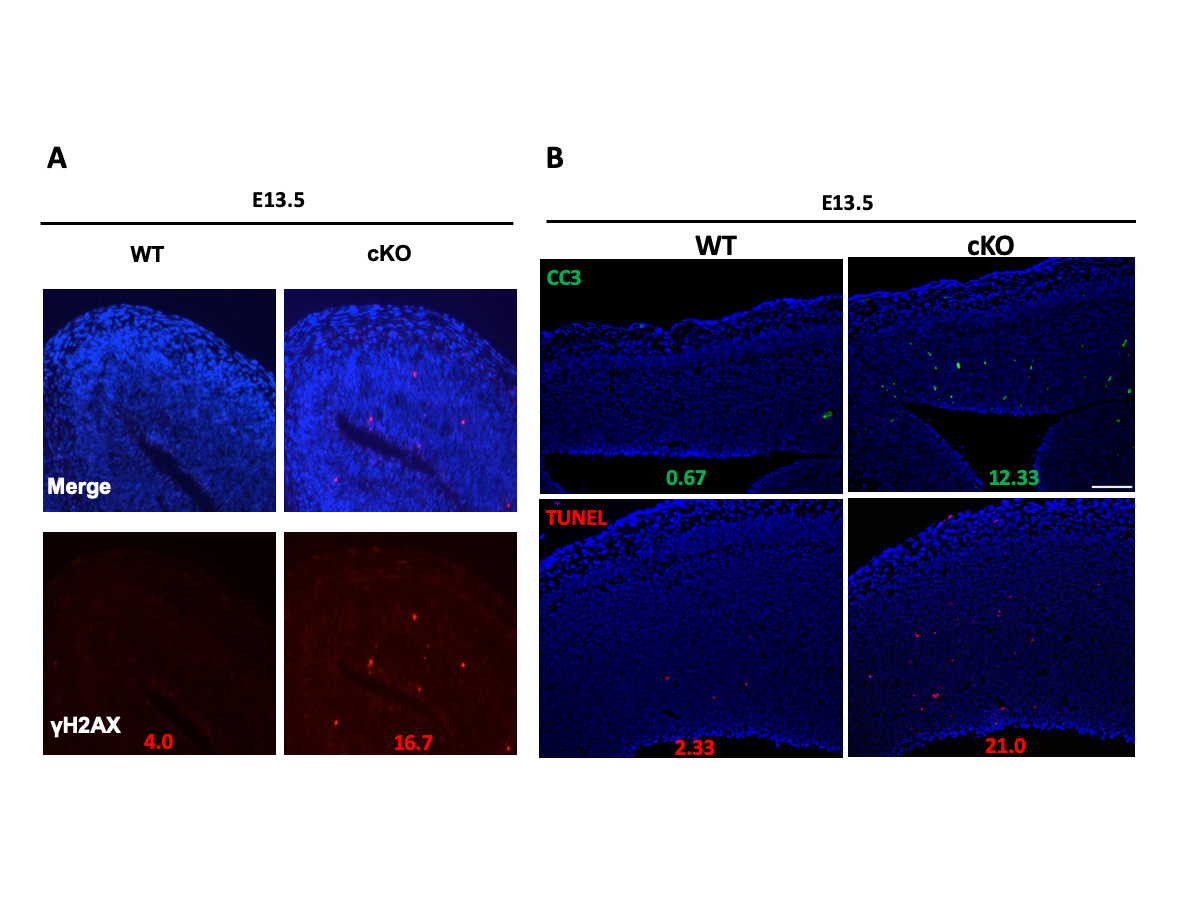


**Supplemental Figure 4: *Snf2h* cKO mice have increased DNA damage and cell death in the developing E13.5 neocortex. A)** Representative epifluorescent images of γ-H2AX^+^ cells (red) in the developing neocortex at E13.5. Nuclei are counterstained with DAPI (blue). The mean number of γ-H2AX+ cells is shown at bottom. **B**) Representative images of cleaved caspase-3^+^ (CC3; green, top panels) and TUNEL^+^ (red, bottom panels) cells at E13.5. The mean number of CC3^+^ and TUNEL^+^ cells is shown at bottom. Scale bars,100 µm. Related to Figure 3.


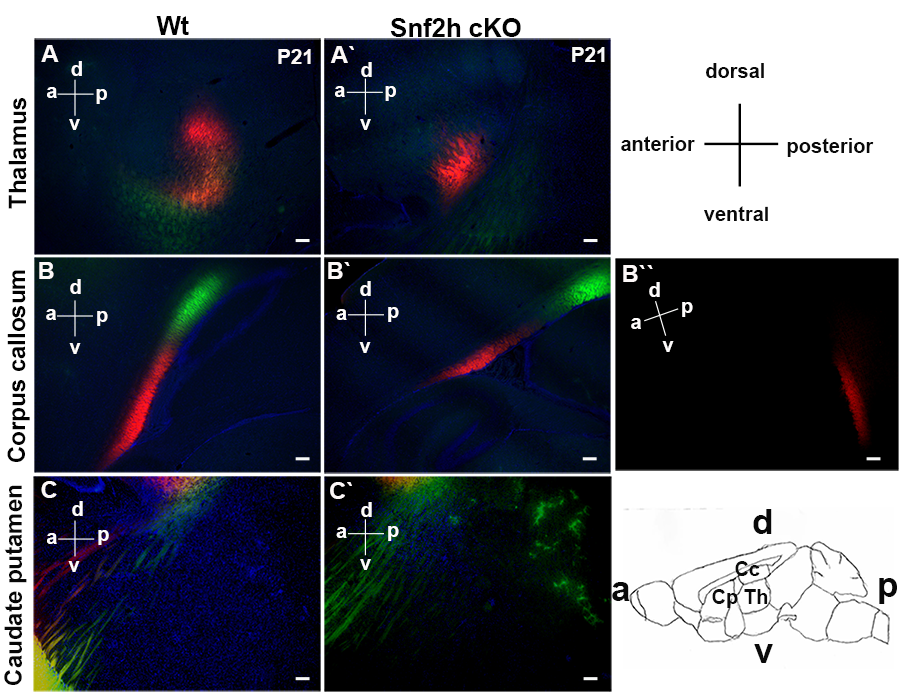


**Supplemental Figure 5: Mapping of cortical projection output in *Snf2*h cKO mice*.***

P21 brains showing neuronal diffusion of both tracers (Dil and DiA) after 6 weeks of incubation in darkness. In comparison with the control littermates, mutant mice show a severe reduction of Dil+ (red) projections in the thalamus (compare A to A’), and also in the CC (compare B to B’-B”). DiA+ (green) projections through the CC were almost absent in more affected animals (B’’). Interestingly, aberrant DiA+ (green) projections were observed in the mutant brain in the caudate putamen (arrows in C-C`). Rightmost top panel shows the relative orientation of the dorsoventral (d and v) and the anteroposterior (a and p) brain. A schematic representation of the whole brain in a sagittal section is shown at rightmost bottom panel to highlight the corpus callosum (CC), thalamus (Th), and caudate putamen (Cp). Scale bars, 50μm. Related to Figure 5.


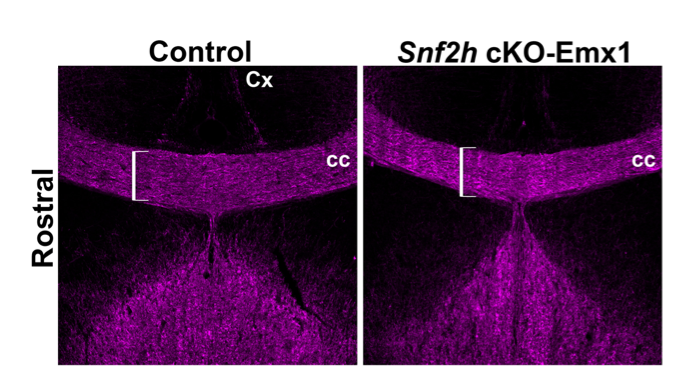


**Supplemental Figure 6: Rostral corpus callosum is largely unaffected in *Snf2h* cKO mice**.

Confocal Z-stacks of rostral coronal sections through the CC of mutant and control mice immunolabeled for myelin-associated glycoprotein (MAG) (magenta), a marker of myelinated neuronal axons. Note that there is no reduction of MAG^+^ axonal fibers in the rostral brain (brackets) compared to the medial and caudal brain regions. CC, corpus callosum; Cx, Cortex. Scale bar, 100μm, n=4 mice per genotype. Related to Figure 6.

**Supplemental Table 1. List of primers used for RT-qPCR.**

| **Target Gene** | **5' to 3' Sequence** |
| --- | --- |
| **Smarca5-F** | GACACCGAGATGGAGGAAGTA |
| **Smarca5-R** | CGAACAGCTCTGTCTGCTTTA |
| **Smarca1-F** | TGCTACAAATGATCCGTCATGG |
| **Smarca1-R** | GCGTTCTCGTTTAGGAGGTTCA |
| **Tbr1-F** | GCAGCAGCTACCCACATTC |
| **Tbr1-R** | GTCCTTGGAGTCAGGAAAATTGT |
| **Eomes-Tbr2-F** | GCGCATGTTTCCTTTCTTGAG |
| **Eomes-Tbr2-R** | GGTCGGCCAGAACCACTTC |
| **Satb2-F** | GCCGTGGGAGGTTTGATGATT |
| **Satb2-R** | ACCAAGACGAACTCAGCGTG |
| **mEbf3-F** | CGAAAGGACCGCTTTTGTGG |
| **mEbf3-R** | AGTGAATGCCGTTGTTGGTTT |
| **mFoxG1-F** | GCTGGACATGGGAGATAGGA |
| **mFoxG1-R** | GGTGGTGATGATGATGGTGA |
| **Bcl11b-Ctip2-F** | GACAAGAGCAGTCCACCTCC |
| **Bcl11b-Ctip2-R** | GGGAAACAGGGTGGGAGAAC |
| **Cux1-F** | TGACCTGAGCGGTCCTTACA |
| **Cux1-R** | TGGGGCCATGCCATTTACATC |
| **Pcdh a4** | forward; GATTCAAGGGACAGAGAGG |
| **Pcdh a12** | forward; AGTCTCCCTCCTGTGTTAGG |
| **Pcdh ac2** | forward; CAACAGGCAACTCACCG |
| **Pcdh aCR** | forward; AGAGCAGGCATGCACAGC |
|  | reverse; GACTGTTTGGGGTTGCC |
| **Pcdh ga3** | forward; CTCACAGATTTACTTGAAACGAAAGAAGACC |
| **Pcdh ga7** | forward; GATTTTCAAGAATGTAAGGGTGAAGC |
| **Pcdh gc4** | forward; GTCCACCCTCTGATCTTCTC |
| **Pcdh gCR** | forward; CTGGCGTTTCTCTCAAGCCC |
|  | reverse; CATGGCTTGCAGCATCTCTG |
| **Diap1** | forward; CAGTCTTCTAGAAGCTCTGCAGTC |
|  | reverse; CTCCGAGGCTAGCAGAGATG |
| **Pcdh b2** | forward; CAGGCTGGGTCCACAATTAGG |
|  | reverse; TGCAGCGAGTTCCTCTACCT |
| **Pcdh b3** | forward; TTTGTTTTTCTGGGTGGGTCTC |
|  | reverse; GATCCGTCGCTAAATTGGCTA |
| **Pcdh b4** | forward; AGGTTCCCGAGAATTTCCCC |
|  | reverse; AGTCCAACTTTTTGCTTAGACGG |
| **Pcdh b5** | forward; CAGGCAAGTGATTCCTTTCCT |
|  | reverse; ATTGCTGTTTGTCATGGTCAGA |
| **Pcdh b6** | forward; TCTGGGTTTCAGGGTAGGGG |
|  | reverse; GTCTCCACATCTAGCTGCAAG |
| **Pcdh b7** | forward; GTGCAGGTGTCCGATGTCAA |
|  | reverse; GGAGATTAACGAGGAGAGTGGC |
| **Pcdh b8** | forward; GGTGTGTTCAATTCAGAACGGA |
|  | reverse; AGGTCAGAGACCGTGATTGTG |
| **Pcdh b9** | forward; ACTGCTCTTGAGAATACCAGAGA |
|  | reverse; AGGACGTGAAAATAAGGGTTGG |
| **Pcdh b10** | forward; CCTTGATGCCTTAGTTGCCAC |
|  | reverse; TACGGAGTTGCCTCAAAATCC |
| **Pcdh b11** | forward; GCTCTGGGGCAACTAGATATTC |
|  | reverse; GGCCAGTTCTCCAACCCTG |
| **Pcdh b12** | forward; CACTGCTCTTGATGGCGGAT |
|  | reverse; GCAGAGACCATGACAACTAAGG |
| **Pcdh b13** | forward; TGCAAGACAAGGCAAGTGATG |
|  | reverse; GCATGGCATACTGAATTGATCCT |
| **Pcdh b14** | forward; GGGAGCAACACGGTTCAAAAC |
|  | reverse; GAGGTGACCCACTGTCCATAG |
| **Pcdh b15** | forward; TTACTGCTCAGTCCGCTTACT |
|  | reverse; TGCAGCGCGATAAATCTGAAAT |
| **Pcdh b16^1^** | forward; GTTCTGGGATGGTTTGGAAATGTAC |
|  | reverse; GACCTCGTTGTGTTTGAGCATTG |
| **Pcdh b17** | forward; TCAGGAAAATGCACGCTGTTA |
|  | reverse; GCTGGTAAGAGCAGATAGCATC |
| **Pcdh b18** | forward; AGGCAAGTGCTATTTCTCTTCC |
|  | reverse; TCACAAACCACCCTAGTTCTCT |
| **Pcdh b19** | forward; ATTGGTCCGAGTGGAGGTCAT |
|  | reverse; TGGAACCGTCACTTCATAGAACA |
| **Pcdh b20** | forward; TCTTTGCGCTATTCTGTAGCAG |
|  | reverse; GATCCAGTCGCTCATTCAGGA |
| **Pcdh b21** | forward; AGCAGCGAACCCTGTATATTGC |
|  | reverse; TTCCTGGAGTAGCACTTTCTGA |
| **Pcdh b22** | forward; AACTATGGTAGGCAACCAGATGATC |
|  | reverse; GAATACAGAGAGCGAAATGAGACG |
